# Comparative analysis of search approaches to discover donor molecules for organic solar cells

**DOI:** 10.1039/d4dd00355a

**Published:** 2025-08-13

**Authors:** Mohammed Azzouzi, Steven Bennett, Victor Posligua, Roberto Bondesan, Martijn A. Zwijnenburg, Kim E. Jelfs

**Affiliations:** a Department of Chemistry, Imperial College London White City Campus W12 0BZ London UK K.jelfs@imperial.ac.uk; b Laboratory for Computational Molecular Design (LCMD), Institute of Chemical Sciences and Engineering, Ecole Polytechnique Federal de Lausanne (EPFL) 1015 Lausanne Switzerland Mohammed.azzouzi@epfl.ch; c Department of Computing, Imperial College London London SW7 2AZ UK; d Department of Chemistry, University College London 20 Gordon Street London WC1H 0AJ UK

## Abstract

Identifying organic molecules with desirable properties from the extensive chemical space can be challenging, particularly when property evaluation methods are time-consuming and resource-intensive. In this study, we illustrate this challenge by exploring the chemical space of large oligomers, constructed from monomeric building blocks, for potential use in organic photovoltaics (OPV). For this purpose, we developed a python package to search the chemical space using a building block approach: *stk-search*. We use *stk-search* (GitHub link: STK_search) to compare a variety of search algorithms, including those based upon Bayesian optimisation and evolutionary approaches. Initially, we evaluated and compared the performance of different search algorithms within a precomputed search space. We then extended our investigation to the vast chemical space of molecules formed of 6 building blocks (6-mers), comprising over 10^14^ molecules. Notably, while some algorithms show only marginal improvements over a random search approach in a relatively small, precomputed, search space, their performance in the larger chemical space is orders of magnitude better. Specifically, Bayesianoptimisation identified a thousand times more promising molecules with the desired properties compared to random search, using the same computational resources.

## Abstract 2

*stk-search* employs multiple search algorithms, including Bayesian optimization, to systematically navigate the chemical space of molecules constructed from modular building blocks.

## Graphical abstract

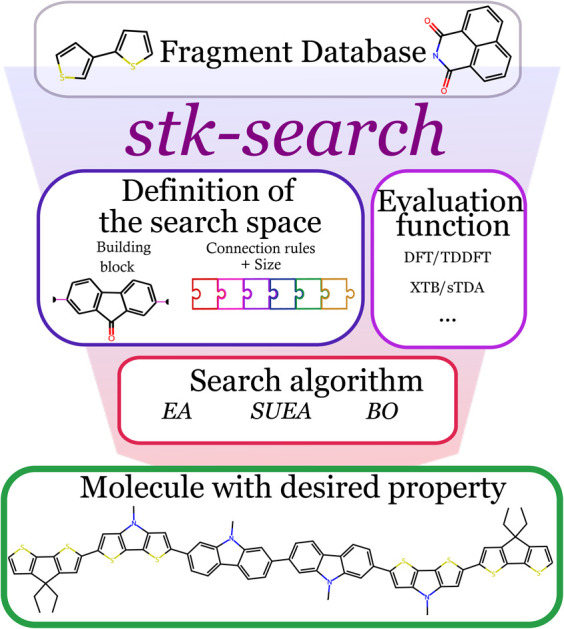

## 1 Introduction

Organic semiconductors have emerged as a versatile class of materials, holding promise for various optoelectronic applications, including in flexible screens, electronic devices, and transparent, lightweight photovoltaic systems.^1,2^ However, the successful adoption and integration of organic molecules into targeted devices heavily relies on the discovery of new molecules with optimal optical and electronic properties, as well as considerations of their synthesis cost, solubility in green solvents, and chemical and physical stability.^3^

Exploring the vast chemical space of molecules for organic electronics presents a significant challenge. With an abundance of different molecular structures available for investigation, even slight changes in the chemical composition can profoundly impact the properties of these materials. Among the various approaches to explore this chemical space, a building block strategy is highly attractive.^4,5^ By constructing larger molecules from smaller building blocks, we gain the ability to define a chemical space solely based upon combinations of these building blocks.^6^ This combinatorial definition of the chemical space renders it more manageable for exploration. Thus, the chemical space can be enumerated and is constrained by the size of the building block library and the number of building blocks in the oligomer molecule. With the defined chemical space, the next step is to evaluate the potential of the molecules for the targeted application. Ideally, we would determine a molecule's properties by synthesising the molecule in the laboratory and measuring its characteristics. This step is time and resource expensive, and unfeasible at a large scale considering the size of the chemical space. To reduce the cost of the search, we can use computational evaluation to determine a smaller number of potentially promising molecules.

A computational evaluation requires two steps: assembling the building blocks to construct a molecular model, and a second step in which the properties of the molecule are predicted using computational chemistry methods. Several tools are available to build molecules from building blocks, offering good starting geometries for the constructed molecules.^7^ Specifically, we consider in this work for this purpose our software package *stk*, which offers automated assembly and geometry optimisation.^8^ The next step is to evaluate the potential of the molecule for the target application. In the literature we can distinguish between property based evaluation functions, which directly relate to relevant properties of the molecule such as optoelectronic properties (*e.g.*, excited state energy, ionisation potential),^9^ and accessibility based evaluation functions,^10^ that focus on the synthesisability of the molecule and its ease of use for the application of interest. For example, in the case of organic electronics, we are interested in how easily we can deposit the molecule on a surface to form a film.

Evaluating optoelectronic properties typically requires computationally expensive quantum chemistry calculations that can take hours to days.^11^ Consequently, a brute force search of the entirety of the possible chemical space quickly becomes unfeasible. We therefore require efficient search strategies for navigating the vast chemical space to find the most promising systems. One approach that has been explored is the development of machine learning models that alleviate the use of expensive quantum chemical calculations.^12–16^ These models can be used as an initial filter in a high-throughput approach to reduce the size of the chemical space of interest to a more manageable size.^14,17^ The application of statistical models for molecular discovery is, however, limited by the availability of representative datasets upon which to build the statistical model. This limitation can result in statistical models with low accuracy and biased predictions, which could hinder the discovery effort. Another approach relies on the use of adaptive strategies, which selectively explore the search space, and suggest the most promising candidates based on prior knowledge.^18^ These adaptive strategies often incorporate domain-specific information, historical data, or heuristics to guide the search process effectively. Evolutionary algorithms, as an example, demonstrate the power of adaptation in optimisation. These algorithms mimic the process of natural selection, iteratively improving candidate solutions to complex problems. By combining variation, selection, and adaptation, they explore the search space effectively.^19,20^ For instance, Greenstein *et al.* employed an evolutionary algorithm, leveraging specified building blocks, to computationally explore the space of potential organic molecular acceptors and donors specifically for organic solar cell applications.^5^

Bayesian optimisation (BO) is another powerful approach for optimising complex, expensive-to-evaluate functions. Unlike evolutionary algorithms, which explore the search space through variation and selection, BO leverages probabilistic models to guide the search efficiently. Specifically, it employs a cheap-to-evaluate surrogate model that approximates the target property of the search strategy and encodes uncertainty about it. Leveraging this information, the system identifies the next optimal candidate for evaluation based on user-defined criteria. BO has gained prominence as an effective approach for guiding chemical and material discovery. BO's advantages lie in sample efficiency, flexibility, and versatility.^21^ For example, Strieth-Kalthoff *et al.* recently used BO to explore an enumerated space of organic molecules for laser applications, showing a considerable improvement in the search efficiency compared to other approaches.^22–24^ When implementing BO for chemical or molecular discovery, the user faces considerable challenges related to the choice of different molecular representation options and the high dimensionality of the representation space. Molecular representations vary widely, from traditional descriptor-based vectors and molecular fingerprints (*e.g.*, Mordred, ECFP) to string-based formats like simplified molecular input line entry system (SMILES), graph-based embeddings used by graph neural networks (GNNs), and even grid or image-based 3D encodings, and each representation comes with distinct trade-offs in terms of interpretability, invariance properties, and computational cost.^25^ Moreover, in BO we define a decision criterion in the form of an acquisition function to determine which point in the search space should be evaluated next. The acquisition function balances the exploration-exploitation trade-off: exploring regions of uncertainty (where the surrogate model is uncertain about the fitness), while also exploiting promising areas (where the surrogate model predicts high fitness).^26^ The optimisation of the acquisition function over the discrete spaces that are particularly relevant in chemical discovery is very challenging.^21,27^

Here, we introduce a Python package, *stk-search*, that can execute a variety of search algorithms within a molecular chemical space. We explored the application of this package and different search algorithms for a use case targeting organic molecules for application in OPVs. We first evaluated and compared the performance of the different search algorithms on a benchmark dataset in the form of a precomputed search space (comprising 30 000 different oligomers) using a variety of metrics. Then, we investigated how the performance extends to searching across the vast chemical space of 6-mers (comprising over 4 × 10^14^ oligomers built from 6 constituent building blocks taken from a library of 274 building blocks). Finally, we analysed the new oligomers and compared them to oligomers present in the benchmark dataset.

## 2 Methods

We first summarise here the overarching search strategy employed in *stk-search*, followed by a description of the distinct search algorithms implemented in the package.

### 2.1. *stk-search* overview

We developed *stk-search*, an open-source Python package, to efficiently search the chemical space of molecules constructed from smaller building blocks. The package leverages our existing *stk* software, used to assemble the models of the molecules,^8^ along with the *BoTorch* package^28^ for Bayesian optimisation and *PyTorch*^29^ in combination with *Torch Geometric* for neural network models.^30^*stk-search* offers Python functions to facilitate the calculation of the molecules' properties using quantum chemistry calculations. The resulting molecular geometries or position matrices are stored in a MongoDB database, alongside the results of the property predictions.

**Fig. 1:**
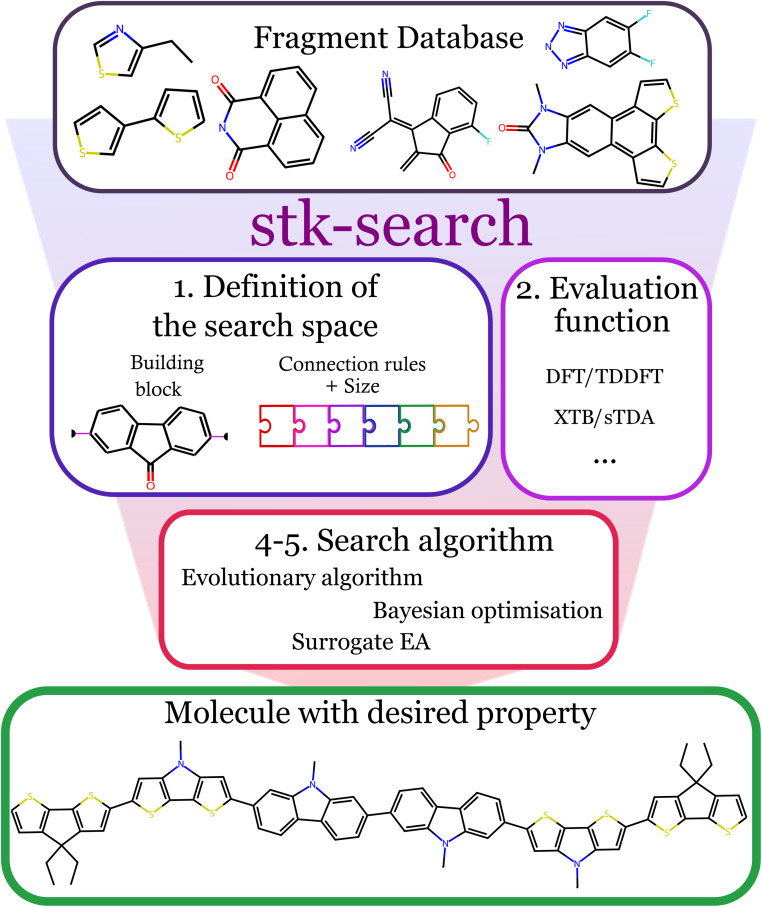
Overview of stk-search. Summary of the procedures implemented in stk-search to explore the chemical space of molecules constructed from building blocks. Starting from a fragment database, we first define the chemical space by generating building blocks with specific connection points, and then establish the size and the connection rules to build the molecules (1). Next, we define an evaluation function where we build the molecules and evaluate their properties using quantum chemistry methods (2). Finally, using an appropriate search algorithm, we can explore the chemical space (4–5) and find molecules with the desired property.

The approach used to search the chemical space within *stk-search* can be summarised by the following steps (Fig. 1):

1. Definition of the chemical search space of the constructed molecules to be explored through the choice of building blocks, the number of building blocks, as well as connection rules for the formation of the larger molecules. Here, the building blocks are molecular fragments with predefined connection points and connection rules (SI 1.a for more details on the search space definition).

2. Establishing an evaluation function that the search algorithm will seek to maximize or minimize in a target molecule. This function can be either a single property, or a combination of properties.

3. Selecting an initial population of candidate molecules from the defined chemical space, using user-defined criteria or random or pseudo-random sampling.

4. Constructing the molecules and evaluating their properties before adding predicted structural and property information for these molecules to the stored database.

5. Using a search algorithm to suggest new molecule(s) to evaluate.

6. Repeating steps 4 and 5 for a user-defined number of iterations or until the computational budget has been exhausted.

### 2.2. Background of the implemented search algorithms

For the four specific types of search algorithms implemented in *stk-search*, we distinguish first between model-free methods and methods that rely on the use of a surrogate model.

In the case of the model-free methods, we considered two examples.

#### 2.2.1 Random grid search (Rand)

A simple approach where the molecules evaluated are randomly selected without replacement from the defined searched space. Without replacement here means that once a molecule has been selected it cannot be selected again.

#### 2.2.2 Evolutionary algorithm (EA)

**Fig. 2:**
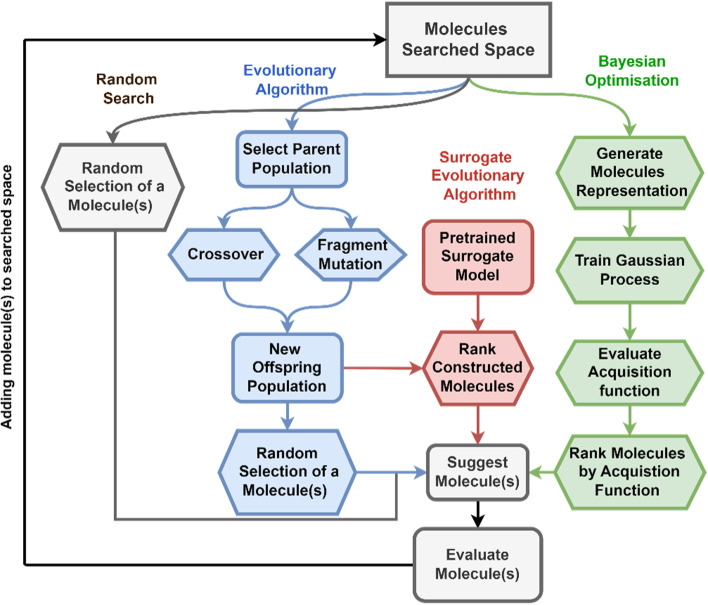
Diagram representation of the different search algorithms implemented in stk-search. The different search algorithms include an evolutionary algorithm (EA), surrogate EA (SUEA), and Bayesian optimisation (BO).

A derivative-free optimisation approach, which explores the vast chemical space following rules that mimic the principles of evolution. One iteration of the EA algorithm consists of, as shown in Fig. 2, the following steps: (i) we select an initial population of pre-evaluated parent molecules based on their evaluation function (often referred to as a ‘fitness function’ in the context of EAs); (ii) from this parent population, a new population of offspring molecules is generated using mutation and crossover operations involving the building blocks; (iii) one or several candidates are randomly selected for evaluation from within the population of offspring molecules. These steps are then repeated for a set number of iterations or until a predefined convergence criteria is reached. While EAs can be powerful approaches to significantly reduce the number of molecules that need to be evaluated before identifying optimal molecules, optimisation of the EA's parameters to increase its efficiency involves adjusting many parameters, including the number of crossovers, mutations, parents, and the number of molecules suggested for evaluation after each iteration.^5,20,31,32^ This parameter optimisation is particularly challenging when the evaluation function is expensive to evaluate, as in the cases relevant to chemical discovery. For this reason, model-based methods are more attractive.

For the model-based methods, we considered two different approaches: methods that use the prediction of a surrogate model without information related to the uncertainty (this is considered a greedy approach), and methods that incorporate a measure of uncertainty in their approach.^33^ Here, uncertainty refers to the variability and potential error associated with the prediction made by the surrogate model. The two model-based methods are:

#### 2.2.3 Surrogate evolution algorithm (SUEA)

A greedy approach that uses efficient surrogate models to approximate the evaluation function and improve the performance of the EA.^34^ We use a surrogate model trained on previously evaluated molecules (before the start of the search campaign) to select a molecule in the offspring population to be evaluated. At each iteration of the search algorithm (Fig. 2), a new parent population is chosen, and the pretrained surrogate model is used to identify the most promising molecule in the offspring population. The molecule with the best predicted property (evaluated using the pretrained surrogate model), will then be evaluated using the chosen evaluation function (expensive evaluation function, *e.g.*, quantum chemistry calculation) and will be added to the population of potential parents.

#### 2.2.4 Bayesian optimisation (BO)

The second category of model-based methods are methods that use the uncertainty of the value predicted by the surrogate model to guide the selection of molecules to evaluate. In this context, the surrogate model provides an estimate of the evaluation function and predicts the uncertainty associated with that estimate. BO transforms the optimisation problem from a costly-to-evaluate black-box function to an acquisition function that is easier to optimise. An example of an acquisition function is the sum of the predicted value and its uncertainty (usually a multiple of the standard deviation or variance of the predicted value), known as the upper confidence bound (UCB). When the UCB serves as the acquisition function, selecting a potential molecule depends not only on the predicted evaluation function but also on our confidence (or uncertainty) in that prediction. One iteration of BO within *stk-search* consists of the following steps: (i) train a surrogate model using Gaussian processes on all or a subset of the evaluated molecules in the search space; (ii) find the molecule(s) in the search space with the highest acquisition function; (iii) evaluate the molecule(s) and add them to the list of molecules the Gaussian process will be trained on. The search algorithm is run for a set number of iterations or until the computation budget is exhausted. For the acquisition function, we can use several acquisition functions implemented in BoTorch, such as expected improvement and UCB. The expected improvement (EI) acquisition function measures how much better a potential solution is expected to perform compared to the current best solution. The optimisation of the acquisition function over the space of molecules is a challenging endeavour. As the acquisition function is a quick to evaluate function, we use an EA to optimise it. In each iteration of the BO search algorithm, we optimisize the acquisition function using an EA. The EA runs for multiple iterations until it converges, and we consider many (in the order of thousands) molecules per generation. The use of the EA here avoids the need to evaluate the acquisition function across the entire chemical space, which is infeasible due to the vast number of molecules.

### 2.3. Surrogate models

For the two model-based methods, we consider models that relate a mathematical representation of molecules to their desired property. The model is trained on prior evaluation of the molecules in the search space. For SUEA, since the model is pretrained before the search process, we can utilize any available model if the inference cost is manageable. This means we can employ traditional machine learning models like random forests for specific molecular representations, or graph neural networks that leverage the position and nature of atoms to construct a representation.^35,36^

For BO, it is essential to use a surrogate model that can be trained quickly and provides an uncertainty measure. Therefore, Gaussian processes are commonly preferred for BO.^21^ The use of Gaussian processes for molecular systems requires the representation of molecules as mathematical objects, such as arrays or graphs.^37–39^ The choice of such representation can strongly influence the performance of the search algorithm, and this choice can be done prior to the search by analysing the existing data we have for the search space. We distinguish here between constructed molecular representations built from a chosen set of properties or molecular descriptors of a molecule's building blocks and learned molecular representations from data available prior to the search campaign. Apart from the choice of representation, for the Gaussian process, the user needs to choose among different kernels that define how the similarity between two molecules relates to the target property. The similarity between two molecules is a function of the representation used, which in this case is often an array representation of the molecules. The different kernels currently implemented in *stk-search* are Mattern, Tanimoto and radial basis function.^39,40^

In *stk-search*, we have incorporated the ability to train and utilize surrogate models based on GNNs. GNNs are powerful models for learning representations from graphical data, making them well-suited for modelling molecular systems. GNNs operate by iteratively updating node features using message-passing operations within the graph structure. The models considered here are 3D based models such as SchNet that take the position matrix of the atoms forming the molecules as input and predict a scalar property of the molecule.^12,15,41,42^ Our implementation relies on the implementation of a graph neural network by Liu *et al.* in their package *Geom3D*.^15^ Since molecules are defined by building blocks and assembly constraints, their atom position matrix is not immediately accessible. To address this, we employ *stk* to assemble the molecules and create an initial geometry. We then use this generated position matrix as input for our model. The initial geometry step is efficient and parallelizable, ensuring it does not impact the search algorithm's performance. Additionally, when molecular geometry significantly influences the evaluation function, we incorporate a training step that relates the specific quantum calculation's geometry to the one initially generated by *stk*.

## 3 Results and discussion

### 3.1. Search space definition

We used *stk-search* on a specific use case; exploring the chemical space of oligomers formed of 6 building blocks, representative of the oligomers and polymers in organic semiconductor applications.^5^ For example, the non-fullerene acceptors used in OPV applications can be split into 3 to 5 different constituent building blocks: ADA or ADA’DA, where A is an electron deficient unit and D an electron rich unit.^43^ For the donor molecules, they can be complex copolymers for which the unit cells can be split into 4 to 6 building blocks.^44^ The chemical space considered here would cover both the space of donors and acceptors currently used and much more. Without introducing any conditions on the building blocks and their positions in the molecule, the number of molecules in the chemical space is *N*^*6*^, where *N* is the number of unique building blocks considered.

As a test case with relevance to the broader organic electronics field, we specifically sought donor oligomers that would work efficiently in a single layer bulk-heterojunction device with the most efficient acceptor in the field (namely Y6 (ref. 44)). We chose here to focus on donor oligomers formed of 6 building blocks as a compromise between loss of accuracy in shortening the oligomer, and the increased cost of screening larger systems. Prior work by some of us showed that the optoelectronic properties of interest here converge with oligomer chain lengths of 6 monomers.^45^ Hence the properties of an hexamer are representative of those of longer oligomers.The compatibility of the polymer with Y6 requires the donor oligomer to have an ionisation potential (IP) 0.1 to 0.2 eV higher than Y6 (which is around 5.65 eV relative to vacuum as experimentally measured^46^) to reduce the energy losses related to the exciton dissociation process and ensure high charge generation yield.^47^ The donor should also absorb strongly in the spectral region where Y6 absorbs little or no light (in the spectral region from 400–550 nm). These oligomer properties can be calculated using density functional theory (DFT), and time-dependent density functional theory (TD-DFT).^48,49^ Specifically, we can calculate the vertical ionisation potential of a single oligomer in vacuum as the difference in ground state energy between the neutral oligomer and its positively charged version. For the optical properties, we limit our calculation to the properties of the first vertical excited state using TD-DFT calculations. We calculate the energy of the first excited state (*E*_S1_) as a proxy for the spectral region where the molecule would absorb, and the oscillator strength of the transition from ground state to first excited state (*f*_osc,S1_) as a proxy for the strength of the transition (*i.e.* the absorption coefficient).^50^

We used the *stk*-generated geometries as initial input, then used the Experimental-Torsion basic Knowledge Distance Geometry (ETKDG) approach in *stk/RDKit* to generate a first geometry.^51^ Next, we optimised the geometry to the lowest energy conformer using GFN2-xTB^52^ and calculated the vertical ionisation potential and electron affinity using the IPEA option in xTB. The optical properties of the oligomers were calculated using sTDA-xTB.^53^ The properties calculated using this combination of methods can be related to the experimentally measured properties using a linear transformation.^54^ Hence this method combination was chosen because it provides a good balance between computational efficiency and accuracy, making it suitable for the high-throughput screening of potential donor molecules for OPV applications.^45^ Inherently more accurate but expensive methods or, indeed, experiments can be used to evaluate the most promising candidates further, but this is out of the scope of the paper, as our main focus is on the comparison between the different search approaches.

To create an evaluation of potential oligomeric molecules that considers the factors mentioned above, we used the following evaluation function that is a combination of the three properties (IP, *E*_S1_, *f*_osc,S1_):

1 *F*_comb_ = −|*E*_S1_ − 3| − |IP − 5.5| + log_10_(*f*_osc,S1_)

We will refer to the value of the evaluation function (eqn (1)) for a molecule as the combined property function (*F*_comb_) of the molecule. The ideal IP is set to 5.5 eV, and the target excited state energy to 3 eV (∼410 nm). The oscillator strength in this case should be maximised. A value of *F*_comb_ above zero means that we have molecules with IP and *E*_S1_ close to the target, and an *f*_osc,S1_ above 1. An oscillator strength above 1 can be related to an absorption coefficient of the film of a value ∼0.02 nm^−1^ (depending on the arrangement of the molecules and other parameters), meaning a film of a thickness of ∼50 nm would absorb all the light at that wavelength.^55^ In the case where *f*_osc,S1_ is zero, indicating a dark first excited state which is detrimental for the use of the molecule as donor in an organic solar cell; the overall score of the molecule in this case is set to a low value of −10. We provide in the SI 1.e details on the computational implementation of the evaluation function.

**Fig. 3:**
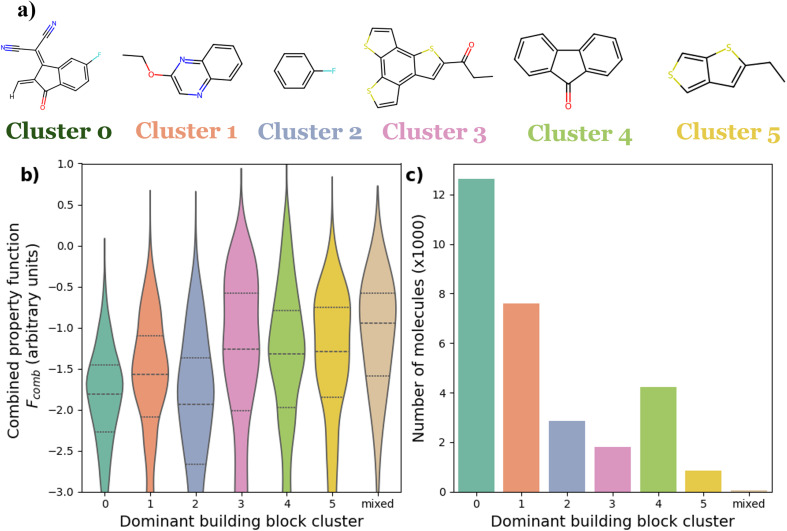
Features of the precalculated search space of 30 000 6-mer molecules; (a) different representative building blocks for 6 different clusters of oligomers; (b) Violin representation of the distribution of *F*_comb_ values of the oligomers with dominant building blocks from different clusters. Here we defined the “dominant building block cluster” as the most frequent building block in the molecule, and when there is no dominant building block cluster, we classify the constructed molecules as “mixed”; the dashed lines split the distribution into 4 quartiles. (c) The frequency of molecules in each cluster of dominant building block clusters.

The chemical space considered in this example, consists of 131 different fragments from the library of Greenstein *et al.*,^5^ these are shown in Fig. S1. We limited the number of atoms per fragment to 30 non-hydrogen atoms. The library of fragments can be combined into building blocks and becomes a library of *N* = 274 different building blocks when all possible routes to combining the fragments are considered. We manually clustered these building blocks by chemical similarity and representative molecules for each cluster are shown in Fig. 3a (Fig. S9 shows the different clusters in 2-dimensional space). These clusters would help us analyse the overall performance of the molecules suggested by the different search algorithms. Cluster 0, for example, is formed of building blocks similar to 3-(dicyanomethylidene)indan-1-one (2HIC), which is an electron withdrawing end-group commonly used to prepare non-fullerene acceptors.^56^ Whereas cluster 4 is formed of three fused-ring building blocks such as fluorene derivatives, which are commonly used in polymer semiconductors.

All ways of combining the 274 building blocks presented above creates a chemical space of *N*^6^ > 10^14^ different 6-mers. In what follows, we first assess the performance of 6 different search algorithms on a constrained chemical space limited to 30 000 randomly precalculated 6-mers from this chemical space. Subsequently we used the different search algorithms to search the larger full 10^14^ chemical space.

### 3.2. Implemented search approaches

Using the four distinct search algorithms described in Section 2, we applied them here with different implementations to create six distinct search approaches. These include the four search algorithms *Rand*, *EA*, BO and *SUEA*. For the BO we consider three different implementations that differ in the representation and surrogate model considered. Here, we consider three different representations to investigate the impact of choosing a molecular representation on the search algorithm performance. Specifically, the six search approaches considered are:

1. Random search (*Rand*): used as our baseline case.

2. Evolutionary algorithm (*EA*); we applied a simple case where five parents are chosen for each generation. Two of the parents are chosen randomly, and the other three are taken as the molecules with the highest *F*_comb_ in the current population. Next, we consider all the mutation and crossover operations possible to generate an offspring population and randomly select a molecule in the offspring population to evaluate. See SI 1.d for more details.

3. Surrogate EA (*SUEA*); We used the same approach as the EA in (2) but using a pretrained model to select the best molecule in the offspring population, rather than randomly choosing one. The pretrained model used here is a deep neural network that relies on the architecture of SchNet.^12^ SchNet was selected due to its well-demonstrated balance between computational efficiency and representational power, making it a practical yet effective choice for large-scale screening tasks. We use SchNet to generate an array representation of the molecules that takes as input the atom types and their coordinates in a specific geometry. This representation is then passed through a feed forward neural network to predict *F*_comb_. Since the properties considered here are strongly affected by the geometry of the molecules considered, we aim to generate a representation of the molecule that is related to their optimised geometry in the ground state at 0 K, *i.e.* the geometry generated following the optimisation using xTB. However, we want to avoid running relatively expensive xTB geometry optimisations to predict the property of interest using the surrogate model. Hence, we include a simple neural network in the training process to map the representation generated using SchNet with the position matrix of the molecules from the *stk*-generated geometry to the representation generated with the xTB optimised geometry. The model is trained on a subset of the precalculated molecules in the database. See SI 1.d more details about the SUEA implementation, and SI 1.e for details on the implementation of the surrogate model used.

4. Bayesian optimisation with a representation generated using the optoelectronic properties of the molecules building blocks (*BO-Prop*). We considered this representation to have potential benefit given that the optoelectronic properties of the larger molecules are related to optoelectronic properties of the building blocks.^25^ Here, we specifically consider a list of the properties of the building block calculated using xTB and sTDA-xTB; IP, *f*_osc,S1_ and *E*_S1_.^52,53^

5. Bayesian optimisation with Mordred descriptors of the building blocks (*BO-Mord*). This considers more general descriptors that are not limited to optoelectronic properties of the building blocks. We built a lower dimensionality representation of the 1200 descriptors available in the Mordred program for each building block and concatenated them to form an array representation of the molecule.^57^

6. Bayesian optimisation with a learned representation (*BO-Learned*), where we used a data driven approach to learn a relevant representation for the property of interest using prior generated data (*i.e.* data generated from previous exploration of the search space and stored in the database). We used the same neural network as for the surrogate model presented in the SUEA in (3) to generate a representation of the molecule. This approach is similar to using a deep kernel to describe the similarity between the molecules for the Gaussian process.^58^ Using this learned representation, we aimed to investigate how the search algorithm would be affected if we used a representation inferred from fitting prior data. Further details are in SI 1.d and 1.e. This approach addresses the limitation of Gaussian processes when dealing with large datasets. It achieves this by using a molecular representation that has been learned from a larger number of training molecules. This representation improves the performance of the Gaussian processes without them needing to be trained on the same number of molecules.^59^

The selection of parameters for various search algorithms can introduce considerable bias, affecting the performance and outcomes in molecular discovery. For example, in an *EA*, the choice of parents and the types of mutation and crossover operations can direct the search towards specific regions of the chemical space, potentially neglecting other promising areas. Similarly, in BO, the selection of surrogate models and molecular representations can result in biased predictions. Furthermore, the use of pretrained models in *SUEA* and *BO-Learned* involves another set of hyperparameters that can significantly influence the search results. Given the complexity of evaluating the overall performance of a search algorithm for a particular task, as discussed in more detail in the following section, we limited our choice of search algorithm parameters to a specific set established through a non-exhaustive parameter exploration.

### 3.3. Assessing the performance of the search algorithms

Assessing the performance of a search algorithm and approach on unknown chemical space is challenging due to the considerable number of parameters to consider. Different search algorithms can perform better or worse for different tasks, and it is often hard to predict their performance on an unknown space *a priori*.^60–62^ Here, the aim of our search campaign was to find new molecules with target properties above a threshold with the least number of quantum chemical calculations having to be performed, given that the quantum chemical calculations are the bottleneck for the high-throughput exploration, and have the largest resource cost.

We first describe the performance of the search algorithm on a restricted benchmark space where we limit the chemical space to 30 000 molecules the properties of which we had previously calculated. Running the search on a benchmark where we know the best solutions helps us to assess how well the search approaches perform. Subsequently we assess the performance of the search algorithm when run over the space with more than 10^14^ molecules.

#### 3.3.1 Benchmark comparison of search algorithm performance

The initial precalculated benchmark space, comprising 30 000 molecules, was selected randomly from the total search space of >10^14^ molecules. Fig. S12 shows a 2D projection of the chemical space using principal component analysis (PCA) and demonstrates that the precalculated chemical space is diverse and samples across the total search space. Fig. 3b shows that no particular building block cluster dominates for either high or low *F*_comb_ values. This is expected as the link between the oligomers structure and these properties is more complex than that.^45,49^

We ran each of the six different search approaches described above (*Rand*, *EA*, *SUEA*, *BO-Prop*, *BO-Mord* and *BO-Learned*) for 400 iterations with an initial random population of 50 molecules. We limited the search to a specific number of iterations to mimic the case where we are constrained by computational resources and can only evaluate a limited number of molecules using the expensive evaluation function.^61^ We are interested in evaluating how fast the search algorithms find the top 1% of the molecules in the dataset (300 molecules in our case). For the search algorithms that required pretraining (of the representation for *BO-Learned* and of the surrogate model for *SUEA*), we hid this top 1% molecules from the training and validation datasets, and then pretrained on a random selection of 20 000 of the remaining 27 700 molecules in the dataset (performance of the surrogate model is presented in Section S5). A comparison between the performance of the search algorithm with a smaller training set of 10 000 molecules is shown in the SI Section 5. To take into consideration the stochastic nature of the search algorithms, we averaged our results over 25 separate runs starting with different initial populations.

**Fig. 4:**
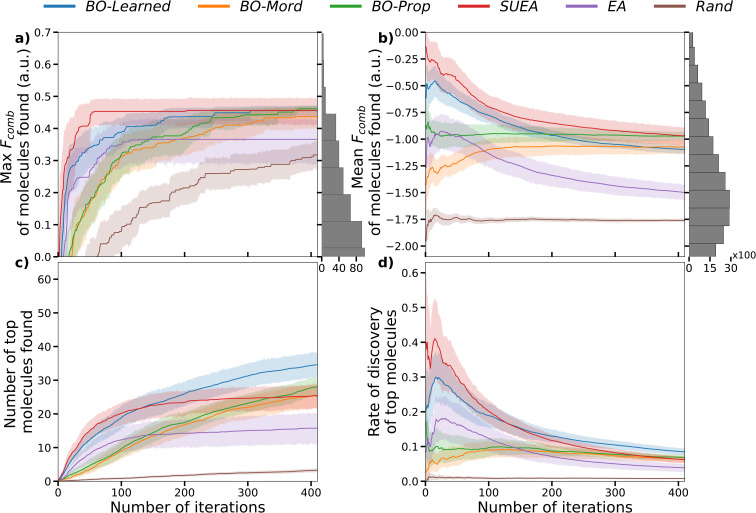
Performance of the six different search approaches on the precalculated benchmark dataset of 30 000 molecules. The solid-coloured lines show the mean *F*_comb_ over 25 runs with different initial populations and the coloured shaded area shows the variance of the *F*_comb_ over those different runs; (a) maximum *F*_comb_ found for an oligomer up to the current iteration. The histogram on the right shows the distribution of the oligomers in the benchmark dataset; (b) mean *F*_comb_ of the oligomers found up to the current iteration; (c) number of oligomers in the top 1% found up to the current iteration (top 1% is 300 molecules); (d) discovery rate of the top 1% oligomers in the dataset, calculated as the (number of top molecules found)/(number of iterations).

The results are shown in Fig. 4. For the six different search approaches, we analysed the best (highest) value of *F*_comb_ found for any oligomer evaluated up to the current iteration (Fig. 4a) and the mean *F*_comb_ for the oligomers at each iteration up to the current one (Fig. 4b). The first metric (shown in Fig. 4a) shows how fast the algorithm finds the molecules with the best properties. The second metric (Fig. 4b) assesses the overall performance of the search algorithm in suggesting molecules that are better than the average molecule in the search space when compared to the baseline. Compared to the baseline *Rand*, the other five search methods consistently identified molecules with a higher *F*_comb_ value after only 100 iterations, outperforming the best result obtained by *Rand* after 400 iterations. *SUEA* manages to consistently find molecules among the top 30 molecules (top 0.1% in the dataset) after less than 100 iterations. *BO-Learned* is the second best and shows a similar rise in maximum *F*_comb_ to *SUEA* in the first iterations, however it only reaches the same maximum value as *SUEA* after 300 iterations. The use of the pretrained representation/model speeds up the performance of the search approaches in finding the best molecules in the dataset. The pretraining also helps the approaches choose molecules with a higher *F*_comb_ in individual iterations. Examination of the molecules selected in the different runs shows that very similar molecules are being selected across different runs of both *BO-Learned* and *SUEA* for the first 50 to 100 iterations (Fig. S18).^63^ After the first 100 iterations, *BO-Learned* started suggesting more diverse molecules. For the other search algorithms, *EA* performs better than *BO-Mord* and *BO-Prop* in the first iterations, but then gets stuck in a region of lower performing molecules and fails to consistently find the top 30 molecules after 400 iterations. *BO-Mord* and *BO-Prop* show a slow but consistent *F*_comb_ improvement over the full 400 iterations, as the surrogate model better learns the search space and begins to perform similarly to *SUEA* and *BO-Learned* after 350 iterations.

Fig. 4c and d focus on exploring how well the search approaches performed at finding the top 1% (300) of molecules, with Fig. 4c showing the number of top 1% molecules found up to a given iteration and Fig. 4d showing the discovery rate of the top 1% of molecules, which we calculate as (number of top molecules found)/(number of iterations). All other search approaches outperform *Rand* by these metrics, as expected. *BO-Learned* performed the best in finding the highest number of top molecules after 400 iterations, approximately 35 top molecules found on average. The discovery rate of top molecules was particularly high in the early iterations for both *SUEA* and *BO-Learned*, before falling over the course of the searches, suggesting the learned representation/model were performing well from the outset. For *BO-Prop* and *BO-Mord*, the discovery rate increases slowly in the first 100 iterations, then it drops slightly later. By the end of 400 iterations, *BO-Mord* and *BO-Prop* find as many top molecules as *SUEA*.

Ideally, you would be able to complete a search such that the top solutions were found regardless of the initial population. This is not yet the case for the 400 iterations here, and some search approaches, in particular *EA*, show much greater variance of outcome dependent on the 25 different initial populations (as exemplified by wider shaded areas in Fig. 4). This emphasises how more effective methods to seed the initial population could significantly improve the search performance.

We extended the evaluation of the search strategies to 800 iterations, allowing each algorithm to explore a larger portion of the chemical space: exceeding 2% of the total benchmark. Compared to the 400-iteration results, a key difference emerged: the random search (Rand) consistently outperformed the model-based approaches after around 700 iterations, identifying molecules with higher *F*_comb_ scores (see SI Section 4.b). This outcome underscores the growing impact of dataset-induced biases over longer search horizons. In particular, model-based algorithms such as SUEA and BO are increasingly constrained by the structural biases in the dataset, favouring molecules composed of frequently occurring building blocks. These biases limit the algorithms' ability to explore under-represented regions of chemical space, especially when the surrogate models and acquisition functions (*e.g.*, Expected Improvement) prioritize candidates that are structurally similar to the majority of the dataset.

Next, we examined the transferability of these observations to the much more demanding task of searching the unrestricted space of more than 10^14^ different 6-mer molecules.

#### 3.3.2 Performance of the search algorithms over the full search space

To compare the six different search approaches over the full search space, we ran each approach with a time restriction of 8 hours for a single run, and with the same computational constraints (30 CPUs and 50 GB of memory). The number of iterations performed by each search run depended therefore on the computational time for calculation of *F*_comb_ for molecules, as well as the computational time needed to suggest new molecules to evaluate. We considered 50 independent runs (with different initial populations) using the same six algorithms used in the benchmark. For *SUEA* and *BO-Learned*, the trained model/representation was the same as for the benchmark study. For each search run, we started from an initial random population of 290 molecules, to which we added the best 10 molecules in the precalculated benchmark space. Adding the best molecules found in the searched space helps ensure that the search approaches start with a better initial population.

**Fig. 5:**
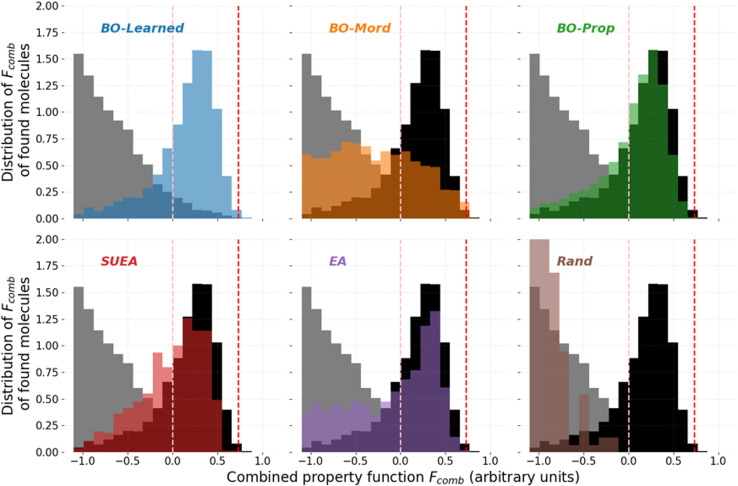
Distribution of the combined property function (*F*_comb_) for the molecules suggested by each search approach. The grey distribution shows the distribution of the benchmark dataset. The black distribution shows the distribution from the BO-Learned search algorithm for comparison. The pink dashed line shows the threshold to have target property above 0 and the red dashed line shows the *F*_comb_ of the best molecule in the benchmark.

Fig. 5 shows the distribution of *F*_comb_ for the new molecules suggested by the different search approaches along the distribution of *F*_comb_ for the oligomers in the database (in grey). We added the distribution of *F*_comb_ of the molecules suggested by *BO-Learned* in black in the other plots to facilitate the comparison. First, compared to the molecules present in the benchmark (grey distribution), all the search approaches apart from *Rand* suggested molecules with higher *F*_comb_. For example, the mean value of *F*_comb_ for the molecules suggested by BO-Learned is around 0.2, where the mean value of *F*_comb_ for the molecules in the benchmark was close to −1.9. Second, we find that *BO-Learned* suggested molecules with overall higher *F*_comb_ compared to the other search approaches. *BO-Learned* suggested the highest ratio of molecules with *F*_comb_ higher than 0 (69% of suggested molecules), next best by this metric was *BO-Prop* (60%), then *SUEA* (54%).

However, if we explore other metrics to compare the performance of the search approaches, it is a different story. Exploring how the approaches performed at finding molecules with *F*_comb_ higher than the ones in the initial benchmark dataset, we found that *BO-Mord* suggested the highest number of better performing molecules (16 molecules, Table 1), twice as many as *BO-Learned*. Although *BO-Learned* uses a representation of the molecules that is better at predicting their combined property, it fails to find molecules better than the ones in the benchmark. *BO-Prop* and *SUEA* each only found one new molecule better than the ones in the initial benchmark, performing as good as the *EA* that is not model-based approach.

**Table 1:** Summary of the metrics to compare performance of the different search approaches over the entire search space. Any approaches with pretraining components were pretrained on 20 000 molecules

Search algorithm	Unique new evaluations	Unique molecules with *F*_comb_ > 0	Rate of discovery of molecules with *F*_comb_ > 0	Molecules found better than benchmark dataset. (outstanding molecules)
BO-Learned	2096	1439	69%	8
BO-Mord	2675	535	20%	16
BO-Prop	3868	2336	60%	1
SUEA	722	394	54%	1
EA	3614	1146	32%	1
Rand	2887	0	0%	0

To assess the generality of the observations made on the first set of runs, we repeated all the search runs, but this time we trained the models/representations on the total calculated dataset at this point of 58 000 molecules, that is the original 30 000 molecules, along with the 28 000 molecules calculated in the preceding runs. The SchNet model was retrained, and this acts as the surrogate model for *SUEA* and the model to generate the representation for *BO-Learned*. For the other search approach, the new set of runs includes the best molecules found in the 58 000 molecules dataset in the initial population. The *F*_comb_ distribution of the molecules in the new 58 000 dataset is shown in Fig. S26. The performance of the search algorithms in the second set runs is shown in Fig. S27 and Table 2. Similar to the first set of results, these findings indicate that the molecules proposed by *BO-Learned*, *SUEA*, and *BO-Prop* have in average higher *F*_comb_ than the ones suggested by the other search approaches. Additionally, in this set of runs, EA identified the highest number of molecules with *F*_comb_ higher better than the best molecule in the starting dataset: 7 new molecules. Followed by *BO-Mord* that found 5 new molecules. This further confirms that the improved performance of the surrogate models to predict the value of *F*_comb_ on the starting dataset reduced the chance of the algorithm to find molecules better than the ones in the starting dataset. This relates to the limitation of the models to extrapolate outside of their training dataset.^64^

**Table 2:** Summary of the metrics to compare performance of the different search approaches over the entire search space. Any approaches with pretraining components were pretrained on 58 000 molecules. The starting dataset here refers to the dataset with 58 000 molecules

Search algorithm	Unique new evaluations	Unique molecules with *F*_comb_ > 0	Rate of discovery of molecules with *F*_comb_ > 0	Molecules found better than starting dataset. (outstanding molecules)
BO-Learned	841	458	54%	4
BO-Mord	1273	406	31%	5
BO-Prop	1799	893	50%	2
SUEA	1037	1004	86%	1
EA	1637	544	32%	7
Rand	1236	0	0%	0

The results of the search approaches consistently show that the algorithms that use a more accurate surrogate model help the search find molecules with in average higher *F*_comb_ (*SUEA*, *BO-Learned*, *BO-Prop*). For *BO-Mord*, the algorithm only suggests 20–31% of molecules with *F*_comb_ > 0 as compared to 69–54% for *BO-learned*. The representation used for *BO-Mord* did not help distinguish molecules by their *F*_comb_ value. EA showed a similar performance at suggesting molecules with *F*_comb_ > 0 (32% in the first and second set of runs). However, the two search algorithms *EA* and *BO-Mord*, showed better performance at finding outstanding molecules, *i.e.* molecules better than the current ones in the starting dataset. This result raises the question of whether a better representation or using a better surrogate model can results in reduced chances of finding molecules better than the ones we had in the starting dataset. This observation is even more important in the case of *SUEA*, as the surrogate model only considers the predicted combined property without any information about its uncertainty. Hence using a pretrained machine learning model can introduce a considerable bias that limits the performance of the search algorithm.

#### 3.3.3 Computational resources needed to run the algorithms

In this part, we discuss the impact of the computational time needed to run the search algorithm on the number of molecules evaluated when using a fixed computational resource. In the two sets of runs presented above, the number of unique new molecules that have been evaluated using the different search approach is different (first column of Tables 1 and 2). Although the same computational resources are allocated to all the different runs, the difference is caused by three aspects; (1) some search algorithms take more computational time to suggest a new element to evaluate. For example, *BO-Learned* needs to generate the learned representation for many molecules before choosing the one with the highest acquisition function. The cost of this optimisation has a significant impact here because the time needed to evaluate a molecule is comparable to the time it needs to optimise the acquisition function. Improving the algorithm used to optimise the acquisition function could reduce this computational cost. (2) The computational time to evaluate molecules can vary from 3 to 20 min depending on the size of the molecule (Fig. S29 and S30 in the SI). (3) When two separate runs simultaneously suggest the same molecule to evaluate, the calculations are run twice. Whereas, if a molecule that has been previously calculated, it will not need to be recalculated. This issue mainly affects the *SUEA*, as the different runs have a higher chance of suggesting the same molecules to evaluate at the same time. Further details about the computation time can be found in the SI Section 8.

Additionally, it is important to note that both *BO-Learned* and *SUEA* depend on a pretrained model, which in this instance was trained on data generated before the search approach began. The process of generating and training this model increases the computational cost of these methods, potentially making them less appealing when there is no initial data available.

#### 3.3.4 Discussion of algorithm performance and surrogate models

Above, we have investigated the performance of six different search algorithms in exploring the chemical space of donor molecules for OPV applications. Our findings indicate that surrogate models significantly enhance the search algorithms' ability to identify superior molecules. The effectiveness of these algorithms in finding molecules above a certain threshold is closely tied to the accuracy of the surrogate models. In essence, more accurate surrogate models are generally beneficial for the search process. This observation aligns with several other studies which emphasize the critical role of model fidelity in guiding molecular discovery.^61,62^

Additionally, we observed that when searching the full chemical space, algorithms with the best surrogate functions (such as *BO-Learned* and *SUEA*) tend to find fewer exceptional molecules compared to searches using less accurate surrogate models (like *BO-Mord*) or heuristic-based searches (*EA*). This discrepancy is due to the surrogate models' limitations in predicting molecules outside their training distribution, which includes these exceptional molecules. Furthermore, the strong performance of *EA* in discovering outstanding molecules is not unique to our study; Tripp *et al.* demonstrated that *EA* can often outperform more complex machine learning methods.^65^ Overcoming this limitation in surrogate models, specifically their reduced generalizability to chemical regions under-represented or absent in training data, could be done through combining different model-based searches with heuristic search such as *EA*.^66^

The failure of the BO based algorithms in finding outstanding molecules is also related to the challenge of accurate uncertainty prediction of molecular properties. Improved uncertainty prediction requires an adapted molecular representation for the target application which is used to compute the distances/similarity between the molecules. In this work, we investigated three different molecular representations, and found that learned representations can outperform expert-curated ones. Furthermore, we demonstrated that achieving strong performance on a benchmark specifically tailored to the task does not necessarily lead to improved identification of exceptional molecules across the entire chemical space.^33,67^

In the context of choosing the best search algorithm for the application at hand, we recommend using a combination of a surrogate model-based approach (*BO-Learned* in this case) with a heuristic based approach (*EA*). This combination would reduce the impact of the bias introduced by the surrogate model or the molecular representation. Coupling this strategy with in-depth analysis of the suggested molecules can help guide the search toward promising regions of the chemical space. To our knowledge, such detailed chemical space analysis is not yet fully automated and would still require a ‘human in the loop'.^68–70^

### 3.4. Analysing the suggested molecules

We have demonstrated the use of six different search approaches to explore the chemical space of donor molecules constructed from various building blocks. In this study, we employed an evaluation function that focuses exclusively on the electronic and optical properties of the molecules, which can be computed relatively quickly using xTB and xTB-sTDA. This choice represents a compromise between relevance and computational efficiency. While more advanced evaluation functions are available within the same package, their use was beyond the scope of this work. Consequently, the molecules identified by the different search approaches can be considered as preliminary candidates for more detailed investigations.

**Fig. 6:**
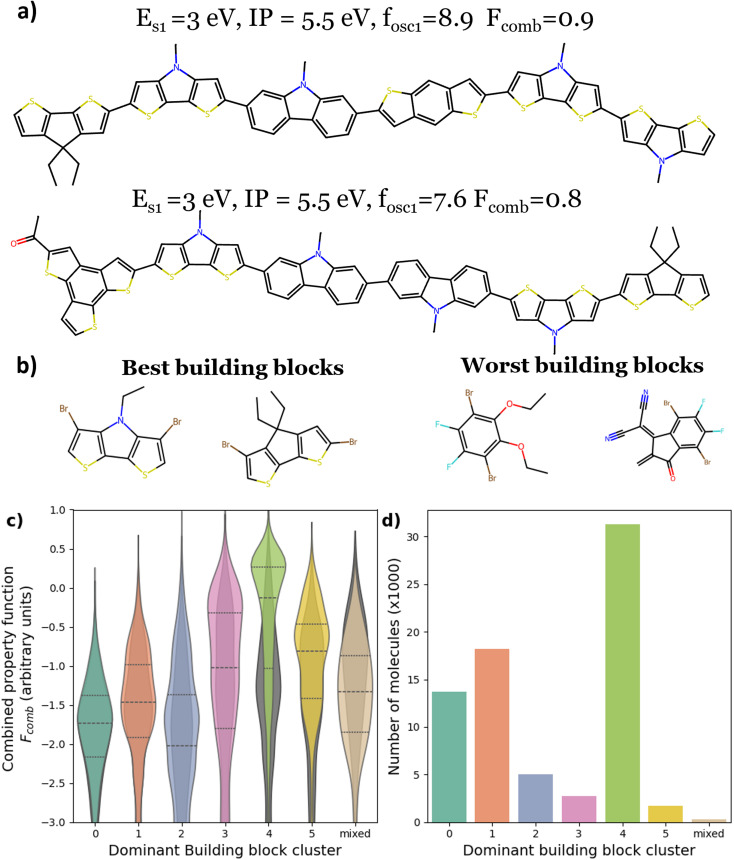
Analysis of all oligomers predicted here across all runs. (a) Examples of the best performing oligomers with their values of *E*_S1_, IP and *f*_osc,S1_. (b) The best and worst performing fragments based on the average *F*_comb_ of molecules containing that fragment. (c) Distribution of the combined property grouped by oligomers with building blocks from different clusters. Here we define the dominant building block cluster for a molecule as the cluster with the highest number of building block present in the molecule. If no cluster is dominant, we label the molecule as “mixed.” The grey violin plots show the data in the benchmark dataset. The lines divide the distribution into quartiles. (d) number of molecules in the dataset grouped by dominant building block cluster. The grey/darker bins show the data in the benchmark dataset.

The *F*_comb_ distribution of all the molecules calculated over all the runs here (78 000 molecules) is significantly different to that of the initial benchmark dataset (Fig. 6c and d, where the grey shadow shows the distribution in the benchmark dataset). In the benchmark dataset, less than 1% of the molecules had *F*_comb_ higher than zero, in the final dataset, more than 22% of the molecules did. This result confirms the performance of the different search algorithms compared to a random search. We also calculated the properties for ten of the best performing molecules using DFT/TDDFT, which confirms that the identified molecules are promising for the targeted application (more details are in the SI Section 9). We have shown that finding molecules with ideal optical and electronic properties that match the requirement established is not particularly hard given these molecules are not rare (at least within the property range predicted by the computational setup used here). The next step would be to build on the findings of this work to establish harder requirements such as the synthesizability of the molecules, the molecular packing, and other properties impacting the exciton lifetime and the charge carrier transport.^71–73^

In Fig. 6c and d, we show the impact of the presence of particular building blocks from a specific cluster on the performance of the molecules. Here, we find that the presence of more than two building blocks from cluster 4 (with the 3 rings fused structures) in the molecule results in a higher *F*_comb_, with a mean above zero. This explains their overwhelming presence in the new dataset, almost 30 000 of the new molecules are in this category. In cluster 4, we can find the benzodithiophene (BDT) structures. BDT and its derivatives are the donor units in most donor polymers that show high power conversion efficiency in OPV devices that use Y6 as the acceptor molecule (*e.g.*, PM6, D18 (ref. 44)). This confirms that our computational approach agrees with the current experimental results and efforts in finding good donor molecules for Y6. Two examples of the best performing molecules are shown in Fig. 6a. Considering only the fragments in cluster 4, we find that among the same cluster, two specific building blocks are better than the rest, these are shown in the Fig. 6b. It is interesting that the BDT unit is not among the absolute best building blocks; for example, 4,4′-alkyl-cyclopenta[2,1-*b*:3,4-*b*′]dithiophene (CDT) was more common in the best performing molecules. Experimentally, the CDT unit is common in donor polymers which performed better with fullerene-based acceptors.^74^ On the other hand, the presence of building blocks from cluster 0 more than once in the molecules results in an overall reduced *F*_comb_.

## 4 Conclusions

We have introduced *stk-search*, a package to run search algorithms over molecules constructed from building blocks, and easily transferable to different use cases. The package considers the definition of the search space based upon the building block library provided, and the connectivity process for constructing molecules from the building blocks. The package also allows the use of different search algorithms including (a) evolutionary algorithms which guide the exploration of the chemical space using rules similar to species evolution (*EA*), (b) An enhanced version of the EA that uses a surrogate model to improve the selection of molecules to evaluate called Surrogate EA (*SUEA*), and (c) an approach that uses the prediction of the molecules performance using a surrogate model as well as the uncertainty on this prediction, namely Bayesian Optimisation (BO). The package also offers different metrics to evaluate the performance of the search algorithms.

We used *stk-search* here to search the space of molecules for application as donors for organic photovoltaic (OPV) applications. We first assessed the overall performance of six different search approaches (including 3 different BO with different molecular representations) over a restricted precalculated benchmark space of 30 000 molecules. In these results we found a strong correlation between the search approaches that suggested overall better molecules considering the combined property function (*F*_comb_) and their ability to find the molecules in the top 1% in the dataset. The explorations with *BO-Learned* and *SUEA* managed to find the best performing results molecules with the least number or iterations. The exploration with these search approaches found the highest number of molecules in the top 1% at the end of the 400 iterations.

When using the different search approaches over a larger search space of >10^14^ molecules, we found that the performance differed significantly to that of the smaller search space. In most cases, the search approaches performed better than in the restricted space. The search algorithms using an efficient surrogate model (*BO-Learned*, *BO-Prop* and *SUEA*), showed a considerable increase in the rate of discovery of molecules with *F*_comb_ above a specified target. On the other hand, the simple *EA*, or the *BO-Mord* using Mordred-based descriptors performed well in finding better molecules than the ones already in the dataset. The added complexity in defining a better representation or using a better surrogate model, only helped guide the search toward overall better performing molecules but fell short of finding molecules with properties better than the original dataset.

These results shed light on how we can use different search algorithms to explore the chemical space of molecules. We have also targeted the question of how we can assess different search algorithms before deploying them. Specifically, we have shown that testing the search algorithm in a benchmark dataset, however close the benchmark is to the task at hand, does not translate to a net improvement when deployed on a much larger chemical space. This discrepancy stems from fundamental differences between a small benchmark dataset and the full search space; most notably, the benchmark's unbalanced representation of the broader chemical space. Therefore, before the widespread use of a new and complex chemical space exploration method, we need to establish proper ways to evaluate them for the specific task. We suggest future work should focus on establishing metrics to evaluate the search space considered and improve the choice of a representative benchmark dataset to compare different search algorithms.

## Author contributions

Mohammed Azzouzi: Conceptualisation, methodology, software, writing of first draft and manuscript, funding acquisition. Steven Bennet: Conceptualisation, software, draft review. Victor Posligua: Conceptualisation, software, draft review. Roberto Bondesan: Methodology, review, and editing. Martijn A. Zwijnenburg: Conceptualisation, methodology, review, and editing. Kim E. Jelfs: Conceptualisation, methodology, supervision, funding acquisition.

## Conflicts of interest

There are no conflicts to declare.

## Supplementary Material

DD-004-D4DD00355A-s001

## Data Availability

The code for running the search algorithms with its different capabilities can be found in the Zenodo record: https://doi.org/10.5281/zenodo.16759043. We also maintain the code in the GitHub link: https://github.com/mohammedazzouzi15/STK_search where we keep the most up to date versions; The code also contains example notebooks to run the experiments conducted in this work, with test data to run them. For the results of the quantum chemical calculation, they are stored in a different record as described below. Calculation data generated during the exploration of the chemical space can be found in the material cloud records: https://doi.org/10.24435/materialscloud:t7-5a. We also provide a notebook to load these data into a local MongoDB database, that would be useful to run the different search algorithms. Supplementary information is available: (1) complete computational methodology and algorithm specifications; (2) detailed fragment and benchmark oligomer databases; (3) comprehensive performance analysis of search algorithms on benchmark datasets; (4) sensitivity analysis of Bayesian optimization parameters and acquisition functions; and (5) full results and computational cost analysis for unrestricted chemical space exploration. See DOI: https://doi.org/10.1039/d4dd00355a.
