# Supplementary material for: Comparative analysis of search approaches to discover donor molecules for organic solar cells
Source: Digit Discov. 2025 Aug 13;4(10):2781–96. doi: 10.1039/d4dd00355a (PMC12379869; doi:10.1039/d4dd00355a)
Supplement: DD-004-D4DD00355A-s001 [file DD-004-D4DD00355A-s001.pdf]

Supplementary Information (SI) for Digital Discovery.
This journal is © The Royal Society of Chemistry 2025

Supporting information Comparative Analysis of search Approaches to Discover Donor Molecules for Organic Solar Cells

Mohammed Azzouzi[1,2*], Steven Bennett[1], Victor Posligua[1], Roberto Bondesan[3], Martijn A. Zwijnenburg[4], Kim E. Jelfs[1,*]

1. *Department of Chemistry, Imperial College London, White City Campus, W12 0BZ, London* United Kingdom.
2. *Laboratory for Computational Molecular Design (LCMD), Institute of Chemical Sciences and Engineering, Ecole Polytechnique Federal de Lausanne (EPFL), 1015 Lausanne, Switzerland*
3. *Department of Computing, Imperial College London, London SW7 2AZ, United Kingdom.*
4. Department of Chemistry, University College London, 20 Gordon Street, London WC1H 0AJ, United Kingdom.

* Corresponding authors

Mohammed.azzouzi@epfl.ch, K.jelfs@imperial.ac.uk

Table of Contents:

1. Computational details: 2
   a. Search space definition. 2
   b. Evaluation function 2
   c. Search algorithm strategy. 2
   d. Search algorithm Details: 3
   e. Model to learn molecular representations 4
2. The fragment database. 7
3. Benchmark oligomer database 12
4. Performance of the search algorithmS on the benchmark dataset 17
5. Impact of training dataset on the performance of the *BO_Learned* on the benchmark dataset 23
6. Impact of choosing different acquisition function on the search algorithm performance 26
7. Results of the search algorithm over the unrestricted space. 27
8. Computation time of searching over unrestricted space. 29

9. References 33

1) COMPUTATIONAL DETAILS:

a. Search space definition.

Our approach relies on first defining the search space through 1) the definition of the fragment library, 2) the way molecules can be connected, 3) restrictions on which fragment can be at different positions of the oligomer, as well as the overall symmetry for the oligomer. Our fragment library is a list of common fragments found in polymers used for optoelectronic applications.[1] We have limited the size of the fragments in this work to fragments with less than 30 non-hydrogen atoms. Then, we can define the building blocks by considering carbon atoms that could form single bonds with a neighbouring fragment. The carbon is then replaced with a "bromine" atom to generate building blocks for the *stk supramolecular toolkit* package. The current approach of defining building blocks can be further expanded by using the full capabilities of *stk* and considering different functional groups for the construction of the large molecules.[2]

b. Evaluation function

The next step in the search approach is to define how to evaluate the potential of certain molecule for a specific application. In the literature we can distinguish between property based evaluation function, which directly relate to relevant properties of the molecule for the target application,[3] and synthesis or accessibility based evaluation functions,[4] that focus on the synthesisability of the molecule and its ease of use for the application of interest. For example, in the case of organic electronic we are interested in how easy we can deposit this molecule on a surface to form a film.

In our case, we focus on property-based evaluation function as detailed in the main text. Following the construction of an oligomer using *stk*, we can use any quantum calculation package to calculate properties of the molecules. All the evaluations of the molecules were saved in a database that can be easily retrieved by the search algorithms, for this we used the *stk* capability of saving the constructed oligomers into a database in MongoDB.[5] Using *stk* we can generate InChIKey for the constructed molecules, that we can then use as a key to save the calculated properties of the molecules in separate databases. In this way, the evaluation method could be a multi-step process, and we can easily retrieve prior calculation outputs for other purposes.

Here, we use the capabilities of *stk* to build the oligomers and generate initial geometries for the quantum chemistry calculations packages. Then we optimise the geometry of the ground state using GFN2-xTB,[6] and calculate the ionisation potential and electron affinity using the IPEA option in xTB. The optical properties of the oligomers are calculated using sTDA-xTB.[7] Afterwards, the properties of the constructed molecules, as well as the xyz coordinates of the optimised geometry, are saved in the database.

c. Search algorithm strategy.

Starting from an initial population of molecules that will form our searched space, we use a search algorithm to suggest a new element in the search space that will be evaluated. The evaluated molecules will be added to the searched space, and we iterate the process for several iterations or until we reach the wall-time of our computational budget. We use a single molecule suggestion after each iteration; however the code can easily be adapted to run a batched search, i.e. where more than one molecule is suggested at each iteration.

When exploring unknown space, we can run several searches in parallel. The stochasticity of the search algorithms and the change in the initial population allows for a better exploration of the search space. Moreover, we use the database to avoid parallel runs re-evaluating the properties of the same molecules through a quantum chemical calculation, which is the time-consuming task in all the search approaches considered.

d. Search algorithm Details:

For our case, where we search over the space of 6-mer for OPV applications, we use the following details for the different search algorithms.

1. *Evolutionary algorithm (EA):* for an iteration we start from a searched space of molecules (current population), which are the molecules that have been evaluated so far during the current search. The searched space is formed of molecules considered in the initial population, and one molecule added after each iteration. We then form the parent population by selecting the top 3 molecules in the searched space and add 2 random ones from the searched space. Then we build the offspring population by performing all the mutation and crossover among the 5 constructed molecules in the parent population. Mutating a molecule in this case means we change a single building block in the molecules with one from the fragment database that satisfies the criteria used to define the search space. The crossover between two molecules means that we take the first X building blocks of the first molecule and the last N-X building blocks of the second molecule to build an offspring, X here is a number from 1 to N the total number of building blocks considered. From the offspring population generated, we select randomly a molecule. The selected molecules will then be evaluated using the defined evaluation function and added to the searched space. Compared to other evolutionary algorithms where we can define different selection algorithms to choose the molecules in the current generation to mutate or cross, we consider here a more straightforward method. We acknowledge that including different selection methods could potentially improve the search performance, however we wanted to show here the performance of the EA in its simplest form.
2. *Surrogate EA (SUEA):* we train the representation learning model described in the next section to learn the combined function values (F_{comb}). We use here a subset of the data we have already calculated in our database (for example either 10,000 molecules or 20,000 molecules). For the benchmark, we hide the top 1% of the oligomers from the training and validation dataset. We use the trained feed forward neural network to predict the combined function. The surrogate model is used to select the element in the offspring population generated using the same approach detailed in the EA section. We select the molecule with the highest predicted combined property among the offspring population for evaluation.
3. For the Bayesian optimisation, we use the RBF kernel with different length scale dimensions as implemented in GpyTorch.[8] We choose the RBF kernel following an initial trial where we tried different kernels such as the Mattern and the Tanimoto-based kernels. In that trial, the RBF kernel performed best, and we decided to use that here. We then use the *BoTorch* implementation to optimise the parameters of the kernel with a normalisation of the input data. Next, we use the Expected improvement (EI) acquisition function as implemented in *BoTorch*.[9] To optimise the acquisition function over the space, we considered the same implementation of the EA described above, apart from the fact that we consider a population of 1000 molecules from the offspring population to calculate the acquisition function at each iteration (compared to one evaluation every iteration for the global optimisation problem). We consider that the EA has converged in this case when the highest value of the acquisition function does not change over 5 iterations.

   We implemented three different specific approaches that use BO:

3.1. Bayesian optimisation with representation from properties of fragment (*BO-Prop)*. We use in this case, optical and electronic properties of the building block to generate a representation of the molecule. These properties are calculated using the same level of theory (xTB and sTDA-XTB). Specifically, we consider the number of atoms, the HOMO and LUMOs, the HOMO-LUMO Gap, the ionisation potential, the electronic affinity, the energy, and oscillator strength of the first 3 excited states. We have 12 properties for each building block which results in a 72-array representation for the 6-mers.

3.2. Bayesian optimisation with Mordred descriptors of the fragment (*BO-Mord)* here we consider for each building block the 1200 different 2D descriptors available in Mordred,[10] to generate a representation of the molecule. We also use principal component analysis to reduce the dimension to 100 which results in a representation of the constructed oligomer in the form of an array of 600 dimensions.

3.3. Bayesian optimisation with a deep Kernel (*BO-Learned*): in this case we represent the constructed molecules using the trained model (the same one used for the surrogate EA). We name this search approach deep kernel since we used a deep neural network to learn a numerical representation of the molecules, which subsequently will be used in the kernel to evaluate the covariance (similarity between molecules). The kernel can be expressed as:

$$K(CM_i,CM_j) = K\big(h(CM_i),h(CM_j)\big) \#(1)$$

Where K here is the RBF (radial basis function) Kernel, and h represents the model used to learn the representation, CM_i,CM_j are two constructed molecules.

e. Model to learn molecular representations.

In this subsection, we present the approach taken to generate molecular representations using deep learning models. This approach is considered to improve the correlation between the representation and the target property. This approach differs from the deterministic methods used in *BO-Mord* and *BO-Prop* in that the molecular representation is learned from a dataset where the target property serves as the label.

We use a deep learning model to learn the representation of the constructed molecules that is most correlated to the properties of interest. We use geometric models such as SchNet to relate the molecules represented as a point cloud with its XYZ coordinates and atomic types to an array representation of the constructed molecules [11, 12]. The geometric models considered use a graph representation of the molecules and use a message passing architecture to embed information's about the environment onto the graph nodes (atoms in this case). Different level of interactions can be considered, and this is one of the characteristics that distinguishes different models. Here, we will only present the use of one model (SchNet), but *stk-search* has other models included such as PaINN or SphereNet, following the implementation by Liu *et al.* [11].

We also included an additional neural network to predict the representation for the optimised xTB geometry of the molecule from the representation of the initial geometry. A detailed representation of the model structure is shown in Figure S1. During the training of the model, we generate the initial array representation of the constructed molecules, using

$$\alpha_{init} = h(CM_{init}) = h(f(BB_i))$$

where h is the geometry representation model, CM_{init} is the constructed molecules in the initial geometry. BB_i is the ith building block, and f is the function used in *stk* to construct the initial molecule. We then use the same method to generate the representation of the constructed molecule using the optimised geometry from xTB as

$$\alpha_{opt} = h(CM_{opt}) = h(f_{xtb,opt}(BB_i)).$$

We use the same geometry representation model to encode the representation of both inputs into an array of 128 dimensions, an array of this size showed the best performance in predicting the property following an initial hyperparameter search. Then we used a feed forward neural network ($FFNN_1$)

$$\alpha_{opt} \sim FFNN_1(\alpha_{init})$$

to transform the array representation of the constructed molecules using the initial geometry to the representation using the optimised geometry. This new representation is then passed through a feed forward neural network to predict the property of interest.

$$Target_{prop} \sim FFNN_2(FFNN_1(\alpha_{init})).$$

The loss function considered to train the model and update the weights and biases is a sum of the mean squared difference (*MSE*) between the two constructed molecules array representation and the *MSE* between the real and predicted target function.

$$L = MSE(\alpha_{init}, \alpha_{opt}) + MSE(Target_{prop}, FFNN_2(FFNN_1(\alpha_{init})))$$

During the evaluation, we would only use the building block to generate the initial constructed molecules representation. This representation can either be used in conjunction with a simple kernel with a Gaussian process for the Bayesian optimisation approach. Or we can use the full model to predict the property of interest and use it as a surrogate model with the *SUEA*.

The choice of the loss function is critical as it directly influences the model's ability to learn and generalize from the data. The *MSE* is a common choice due to its simplicity and effectiveness in minimizing the error between predicted and actual values. However, it is important to consider the potential biases introduced by this choice. For instance, *MSE* assumes that errors are uniformly distributed, which might not be the case in real-world data. Alternative loss functions, such as contrastive loss or weighted loss functions, can be employed to address issues like class imbalance and improve model robustness. In this work, the learning curves with *MSE* were reasonably good not to require the use of other loss functions. We acknowledge however that further improvement on the performance of the surrogate model could be achieved through a different choice of loss function or better optimised learning scheduler.[13]

Computational details: the model was implemented in PyTorch using torch geometric for the graph neural network modules.

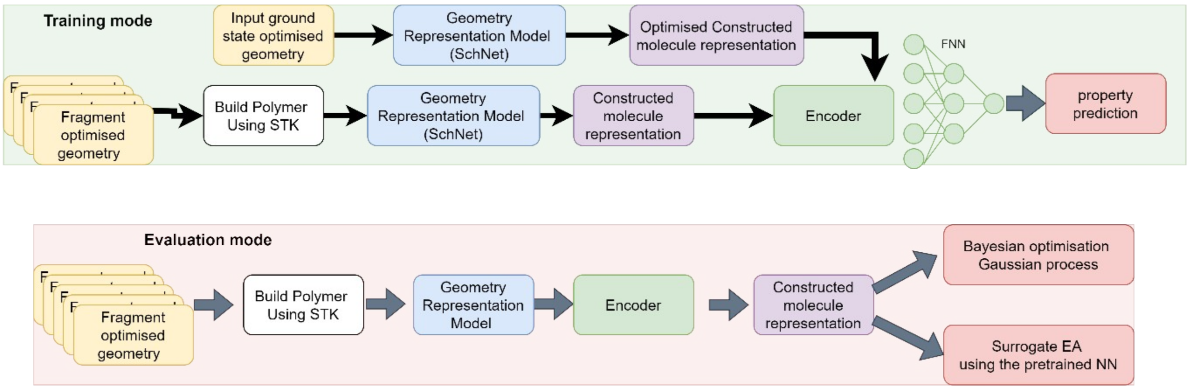

Figure S1 Diagram representing the architecture of the model used to generate the oligomers representation.

2) THE FRAGMENT DATABASE.

The library of building blocks we included are from fragments used in the organic solar cell community[1]. Here, we limit the building blocks to 30 non-hydrogen atoms. To generate a building block from a fragment, we replace a hydrogen atom with a bromine atom to define the connection points.

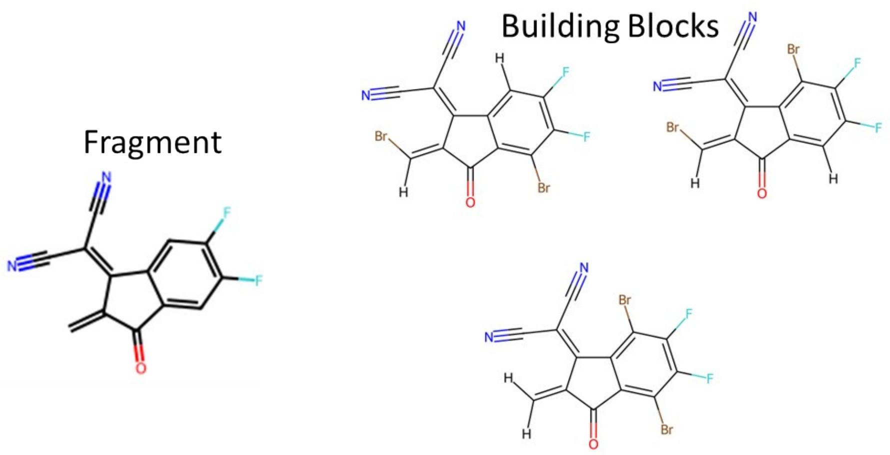

Figure S2 Example of generating a building block from a fragment. Starting from a molecular fragment as on the left, we can define where the two-connection point with other building blocks by replacing hydrogen atoms with a bromine atom.

The list of fragments is clustered using a mixture of manual and automated clustering using the Tanimoto similarity measure based on the ECFP fingerprints.[14] Here we restricted the number of clusters to 6 for ease of interpretation. The list of fragments in each cluster is shown the Figure below. Figure S3 shows the different cluster plotted in 2-dimensional space using the principal component analysis of the Tanimoto distance matrix between the different fragments.

Fragment list per cluster:

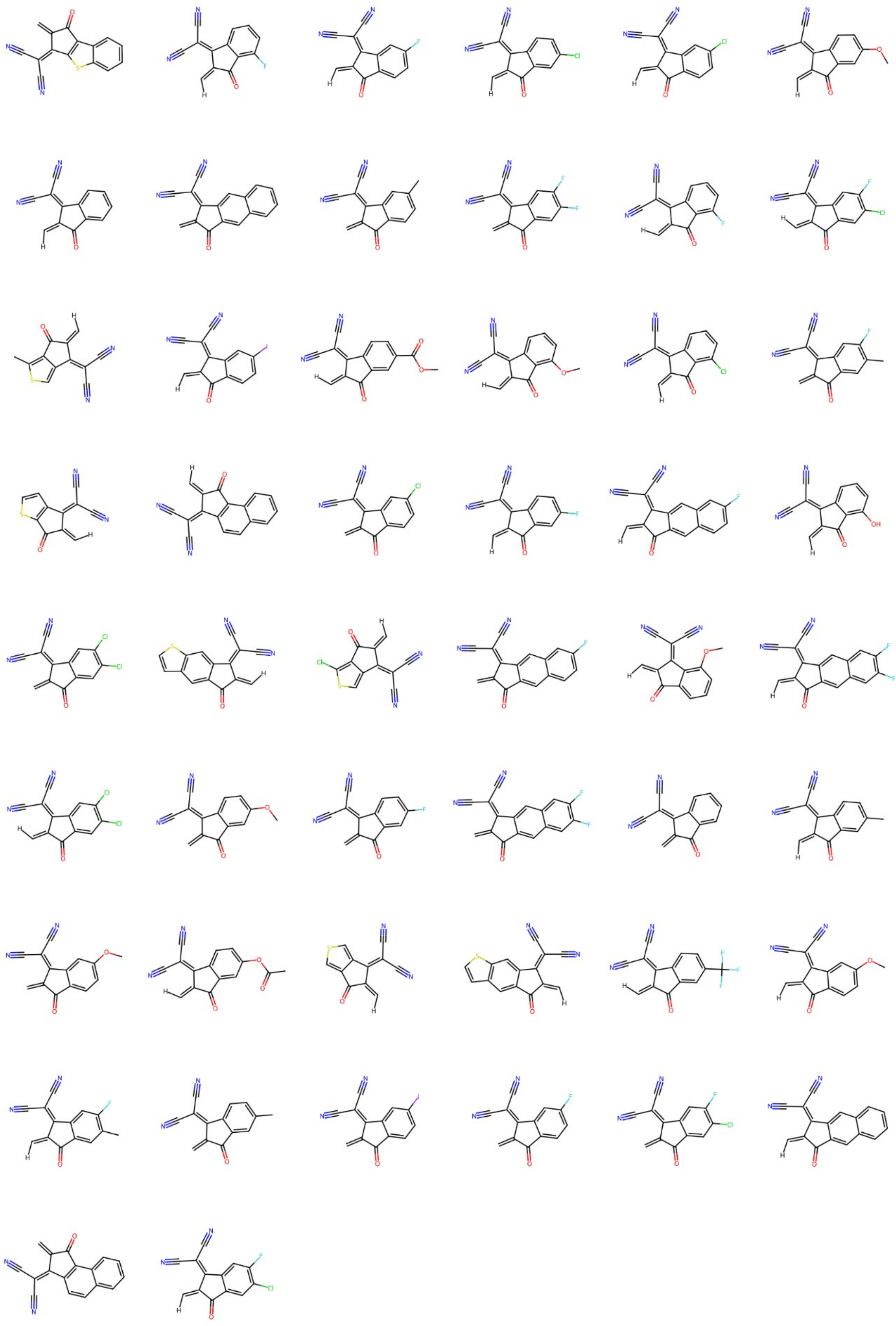

Figure S3fragment in cluster 0.

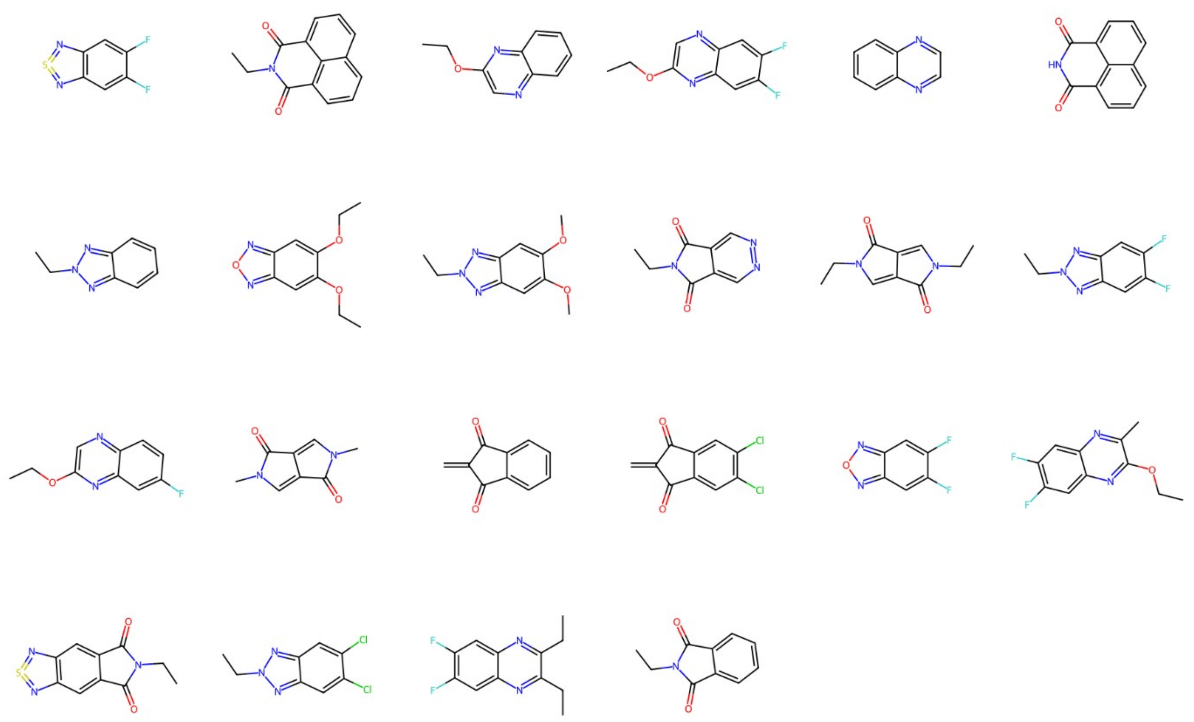

Figure S4 Fragment in cluster 1.

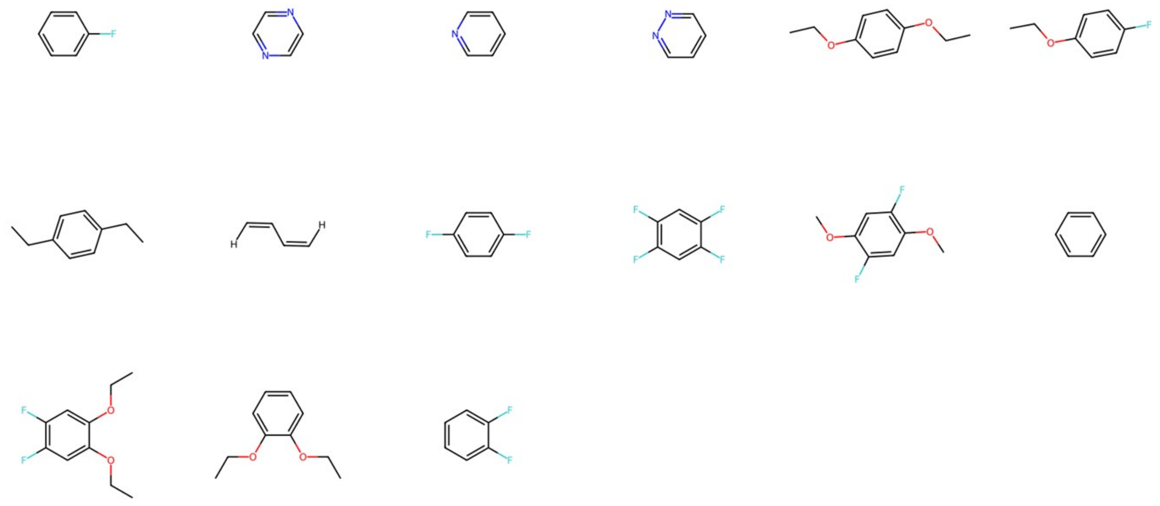

Figure S5 Fragments in cluster 2.

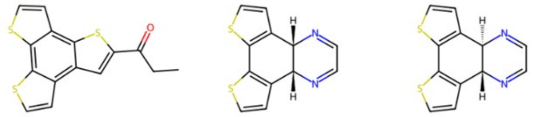

Figure S6fragment in Cluster 3.

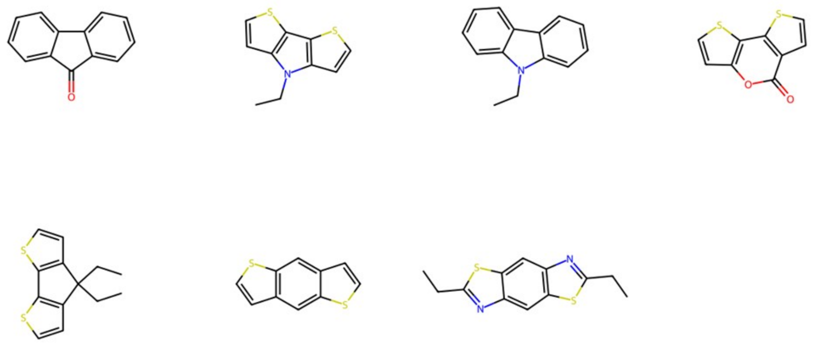

Figure S7 Fragments in Cluster 4.

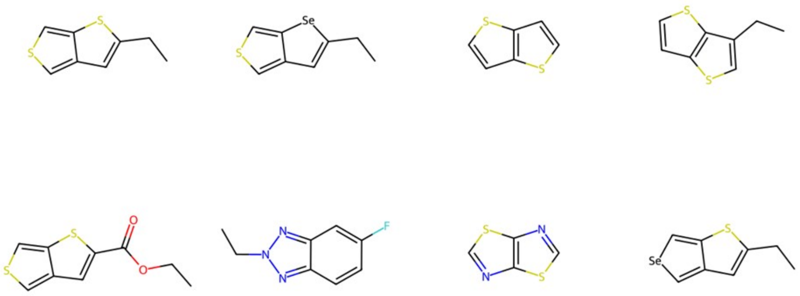

Figure S8 Fragments in Cluster 5.

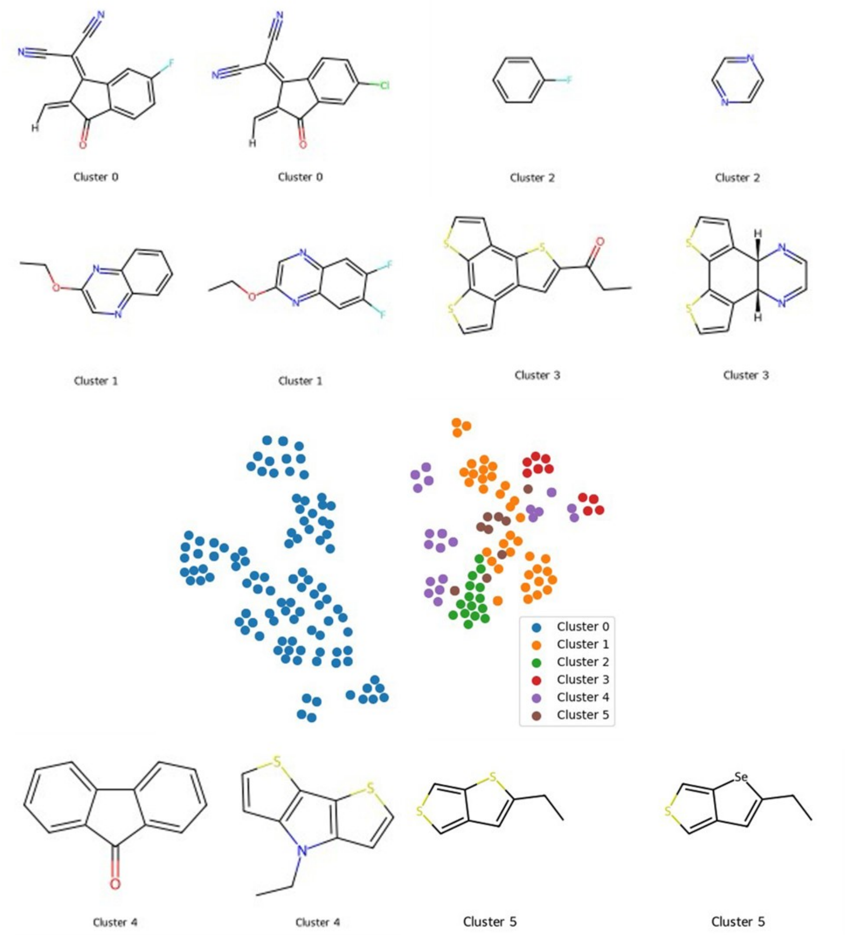

Figure S9 Building block chemical space with representative structures from each cluster. Here the 2D representation of the chemical space is build using the Tanimoto distance matrix between the fragment represented using the ECFP fingerprint.

Figure S10 shows the number of building blocks in each cluster considered. Here building blocks from cluster 0 are dominating the list. This is because there are many different versions of similar structure with fluorine or chlorine replacing the hydrogen atoms.

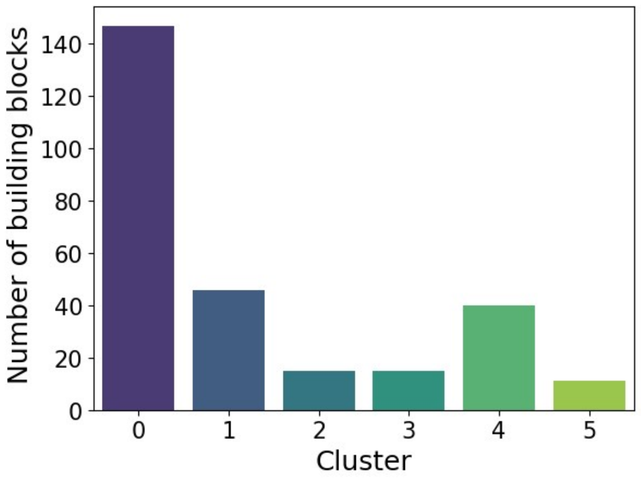

Figure S10 Number of building block in each cluster.

3) BENCHMARK OLIGOMER DATABASE

The molecules present in the benchmark dataset of 30,000 oligomers were randomly chosen from the $>10^{14}$ possible oligomers in the dataset. Figure S5 shows the number of oligomers with building blocks in different positions in the 6-mer. The distribution here is not representative of the proportion of building blocks in each cluster of the building block library (Figure S4). However, when considering the dominant cluster in each oligomer (Figure 2d), the distribution appears to be more representative. This difference can be explained by the very small portion of the full dataset considered in the benchmark (less than 1 in a billion). Hence not all the statistics of the original space are preserved in this benchmark dataset.

The distribution of the different oligomers properties of interest are shown in Figure S7. In this dataset, we find that the ionisation potential is centred around 6.5 eV, and the first excited state energy around 2.8 eV. The oscillator strength is overall low for most of the oligomers, with exceptions having an $f_{osc,1}$ higher than 2. For the combined property, only around 300 different oligomers (around 1%) have a combined property value higher than 0.

Figure S8, shows the distribution of the combined target, by building block in a cluster in the different position of the 6-mer. Here the difference in the distribution does not point toward a specific building block cluster that is considerably better or worse than the others.

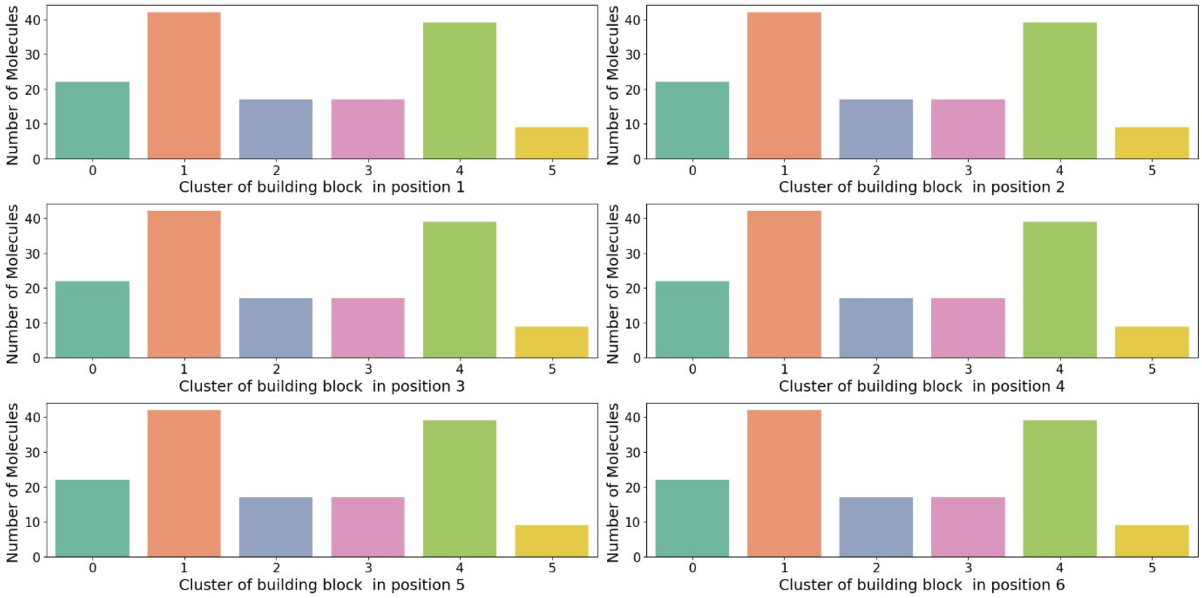

Figure S11 Number of oligomers with building blocks from each cluster in oligomers in the benchmark dataset.

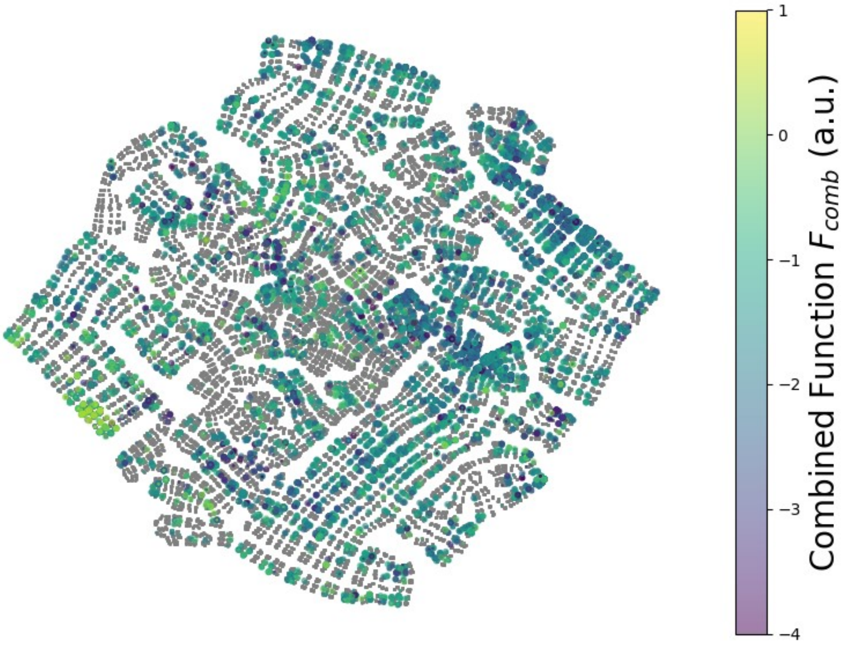

Figure S12 A 2D representation of the chemical space based on a dimensionality reduction of a vectorial representation of the oligomers based where the fragments are represented by their numbered target values. The grey points show the extent of the full space here represented as 6^6 unique points, (the number of fragment clusters is 6, and the total number of unique points would be 6^6.) We used a TSNE dimensionality reduction to produce the 2D representation from the concatenated cluster array. The molecules in the benchmark dataset are here coloured by their combined property values according to the scale on the right of the figure.

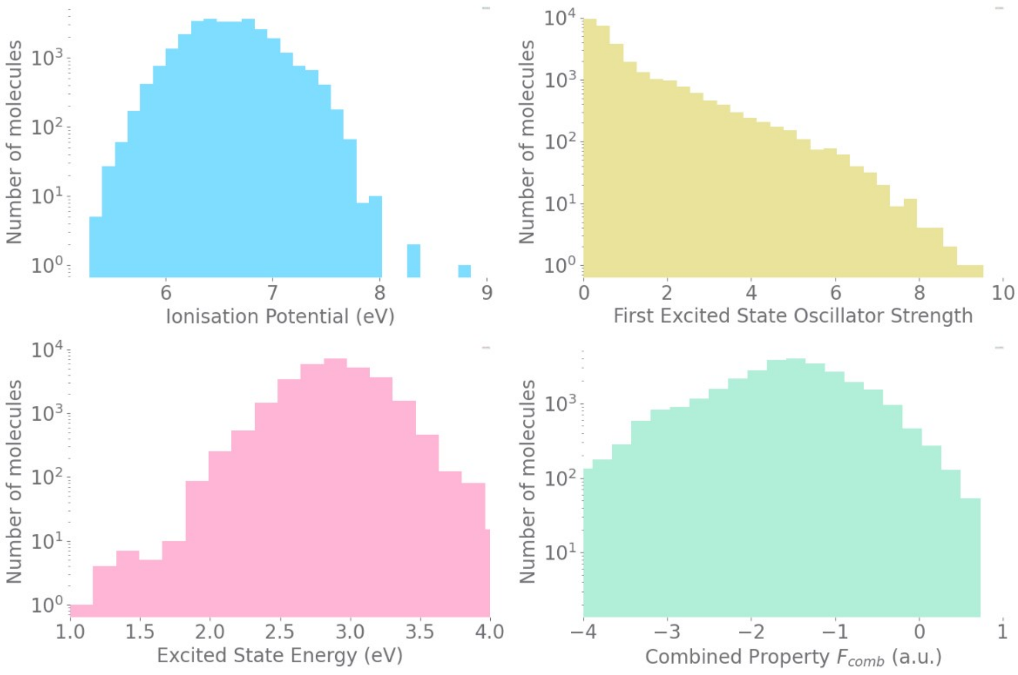

Figure S13 Distribution of the different oligomer predicted properties in the benchmark dataset.

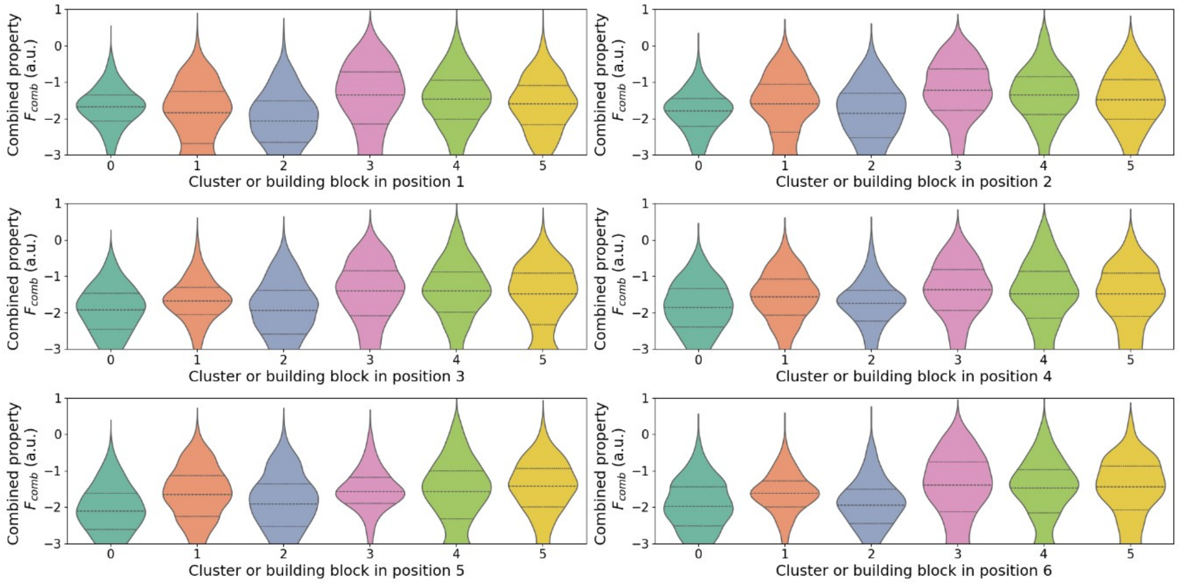

Figure S14 The oligomers' combined property (F_{comb}) distribution with building blocks from different clusters in different positions. The different positions here refer to the position of the building block in the oligomer chain. This Figure shows the distribution of the combined property with the presence of building blocks from the different cluster can impact the distribution of the combined property. For example, in this dataset molecules with building block from cluster 3 in the first position (position 0) have a higher overall combined property (pink violon plot).

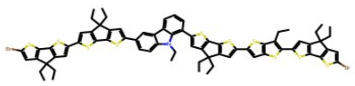

DC 4, CP 0.73, ES1 2.61,IP 5.48, fosc1 8.78

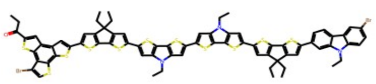

DC 4, CP 0.71, ES1 2.72,IP 5.48, fosc1 7.45

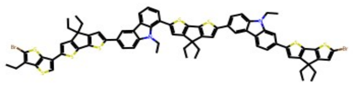

DC 4, CP 0.71, ES1 3.03,IP 5.54, fosc1 5.82

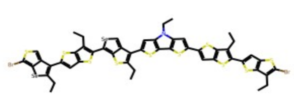

DC 5, CP 0.54, ES1 2.69,IP 5.66, fosc1 7.14

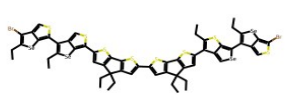

DC 5, CP 0.39, ES1 2.93,IP 5.81, fosc1 5.50

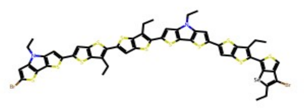

DC 5, CP 0.25, ES1 2.61,IP 5.68, fosc1 4.21

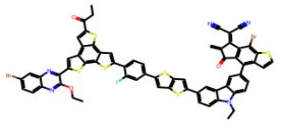

DC mixed, CP -0.07, ES1 3.09,IP 6.23, fosc1 5.03

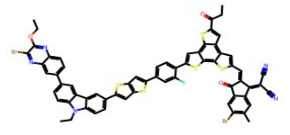

DC mixed, CP -0.20, ES1 2.95,IP 6.30, fosc1 4.27

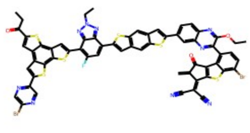

DC mixed, CP -0.26, ES1 2.80,IP 6.24, fosc1 3.79

Figure S15 Example of best oligomers with dominant clusters from cluster 4, 5 and the mixed case. Here DC stands for dominant cluster of building blocks. The dominant cluster is the building block cluster with the highest number of building block in the molecules. Mixed here refer to the case where there is no dominant cluster.

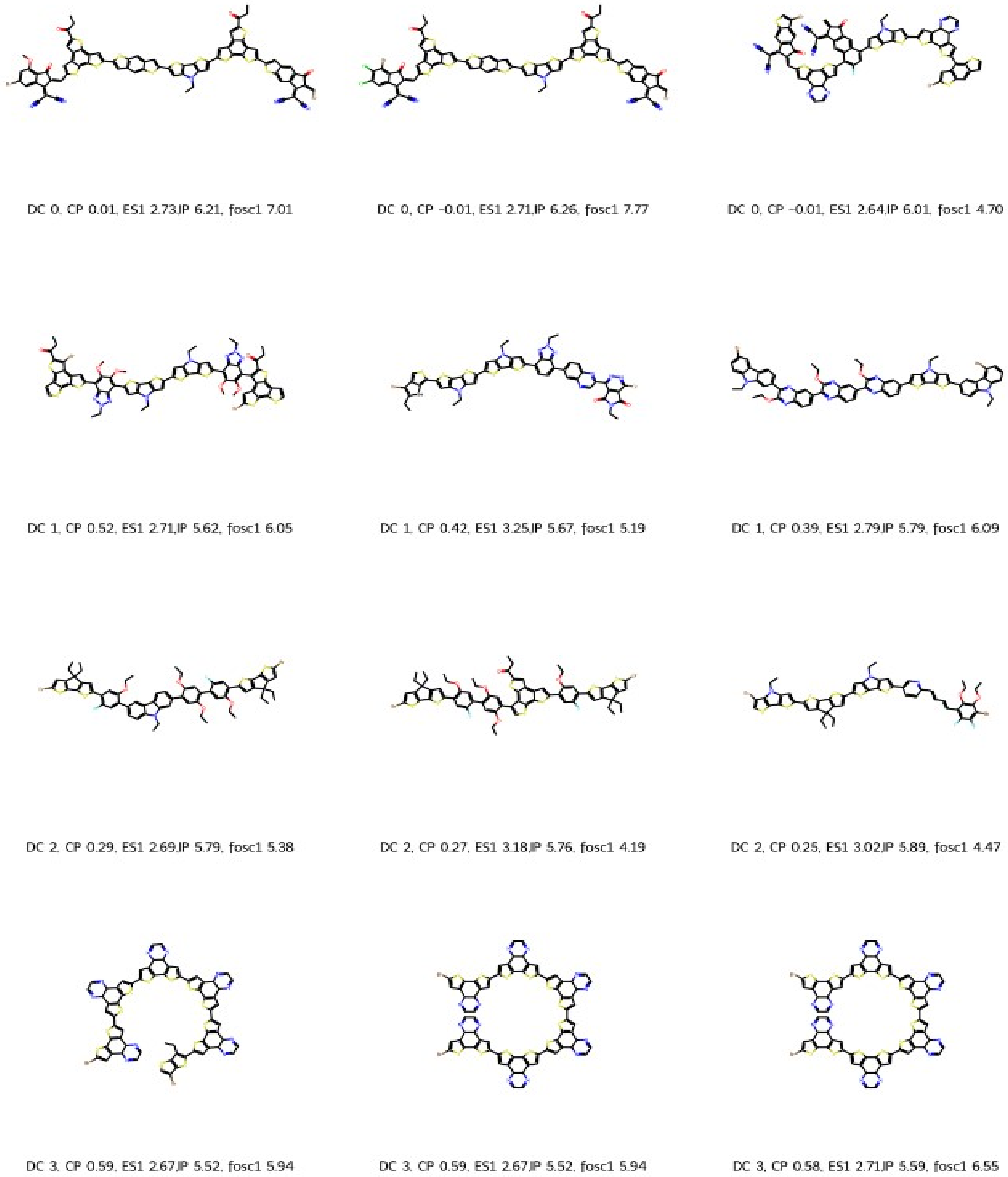

Figure S16 Example of best performing oligomers with dominant building blocks cluster 0,1,2,3. Here DC stands for dominant cluster of building blocks. The dominant cluster is the building block cluster with the highest number of building block in the molecules. Mixed here refer to the case where there is no dominant cluster.

4) PERFORMANCE OF THE SEARCH ALGORITHMS ON THE BENCHMARK DATASET

a. Performance after 400 iterations

To assess the performance of the search algorithms, we introduce a few extra metrics related to the similarity between the molecules suggested during the search. We distinguish between:

1) Similarity of the suggested oligomers to the initial population. This is calculated as the maximum Tanimoto similarity between the oligomers suggested and the oligomers in the initial population [16]. This metric helps assess how the outcome of the search algorithm depends on the initial starting point, and how the initial population impacts the way a search algorithm explores the chemical space.

2) Similarity distribution of the oligomers suggested. This is calculated as the mean similarity between the oligomers suggested by the search algorithm (i.e. considering all the molecules suggested in the previous iterations). This metric helps assess how the search algorithm explore the space. A high value of this metric means that the search algorithm focuses on a specific area in the chemical space.

The assessment of the 6 different search approaches using the two metrics above is shown in Figure S18. First, we consider the similarity of the molecules suggested to those considered in the initial population. Here we find that the *EA* seems to be the most bound to the initial population considered, where the *BO-Learned* shows similar behaviour to the random search. It is worth noting that here we consider the Tanimoto similarity based on the ECFP fingerprint, which are considerable different to the representation considered in the *BO-Learned*. For the similarity between the oligomers suggested by the search algorithms (Figure S10b), the *BO-Learned* and *SU-EA*, suggest very similar molecules after the first few iterations. Due to the more exploratory nature of the *BO-Learned*, it starts suggesting more diverse oligomers after 100 iterations.

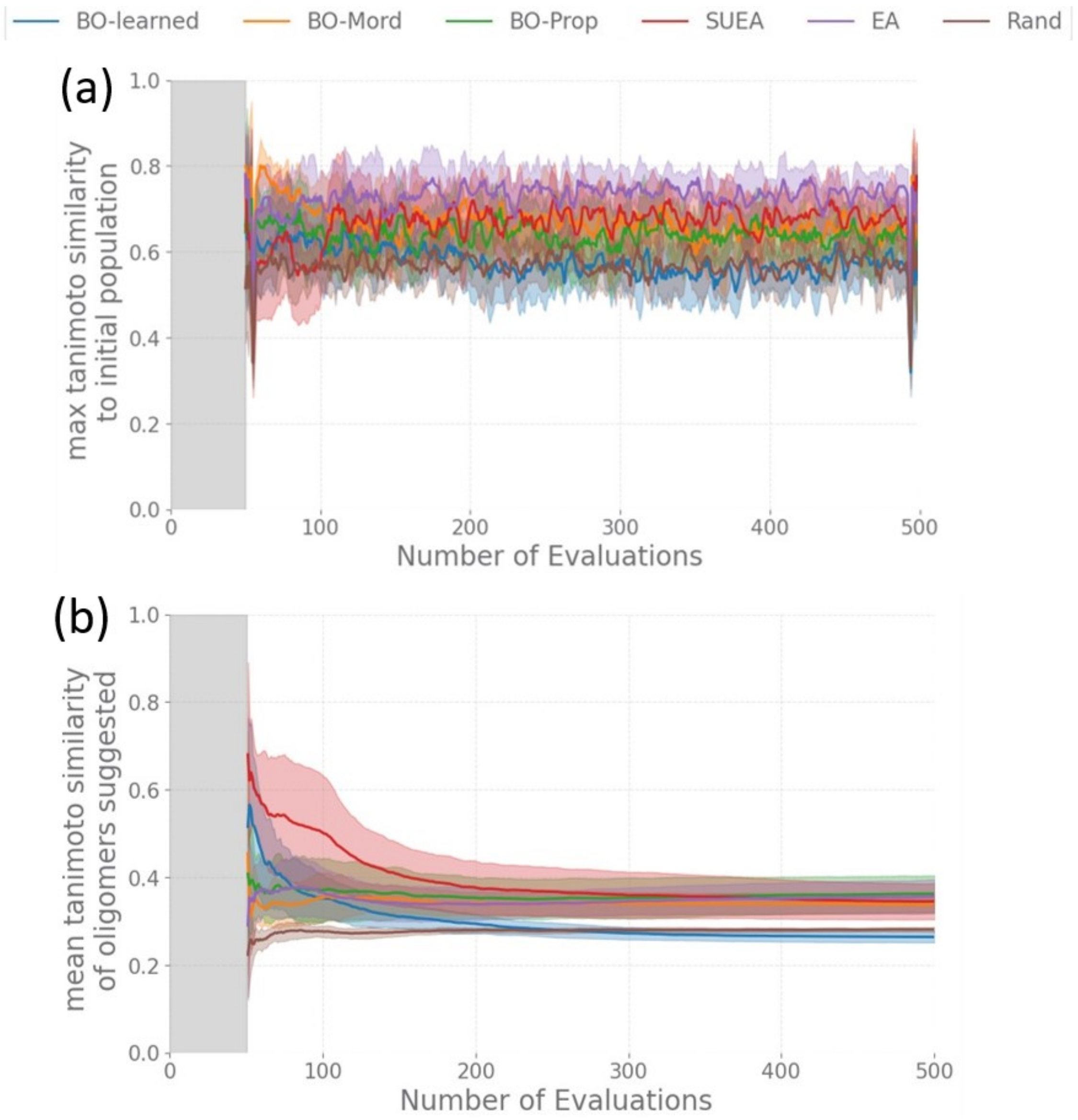

Figure S17 a) maximum Tanimoto similarity of the molecules suggested at the current iteration to the elements in the initial populations of molecules. b) average Tanimoto similarity between the molecules suggested by the search algorithm. The grey shaded area shows the first 50 initial iterations. The colour shaded area around the curves shows the deviation of the metric within 1 variance. The variance is calculated here over 25 independent runs with different initial populations.

In Figure S19, we show a 2D representation of the search space using the different representations considered for the BOs. The figure shows how the learned representation helps smoothing the chemical space and brings oligomers with similar target property closer. The choice of the representation can make the search space to explore smoother and easier to explore, grouping the best performing oligomer in space. Hence the Gaussian process used in the BO will perform significantly better at predicting the performance of the oligomers in the search space. In orange scale, we show in Figure 5, the elements suggested by a *BO-Learned* search. In the space of learned representations, we can see that the search algorithm mostly suggests elements that are close to the best ones seen in the dataset. Then it starts exploring the space more where it has higher uncertainties. The learned representations are however not perfect for the top 1000 oligomers not considered when learning the representation, as shown by the performance of the predictive model on those data.

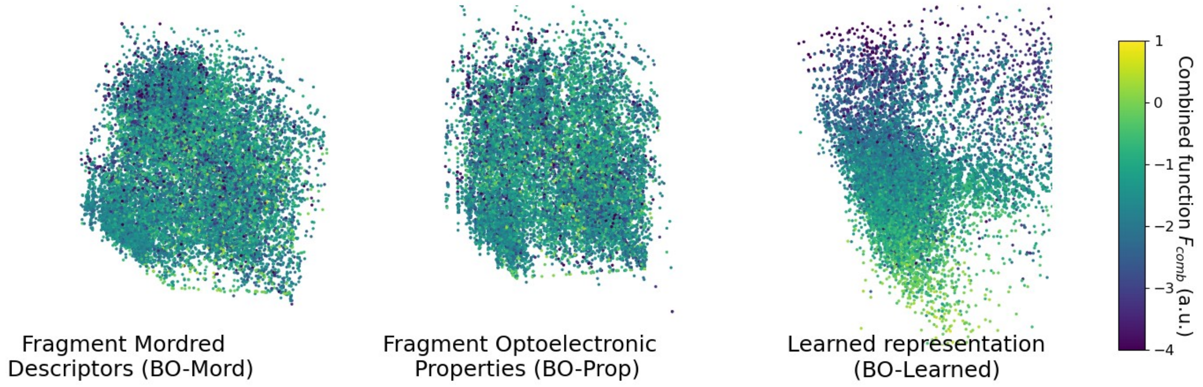

Figure S18 A 2D representation of the chemical space of precalculated oligomers. The representation is based on a PCA of the used representations for the different BO search algorithm. The evolution of a search algorithm using BO with the learned representation and an expected improvement acquisition function is shown in orange scale. The scale goes from light to dark with the number of iterations. The other colour scale shows the combined target value.

In Figure S20 and S21, we look at the performance of the search algorithm considered across the set of 25 runs. Here we consider the distribution of the combined property for the unique oligomers suggested by each algorithm. The oligomers suggested by the random search have a similar distribution to the oligomers in the benchmark (shaded histogram in the plots). In this limited search space, we find that the *BO* and *SUEA* search algorithms, suggest overall elements with higher target property than the random search. It is hard to distinguish the different BOs in these graphs, as they all show similar distributions. The *EA* shows the least difference with the *Random search*, which explains its limited performance in this search space.

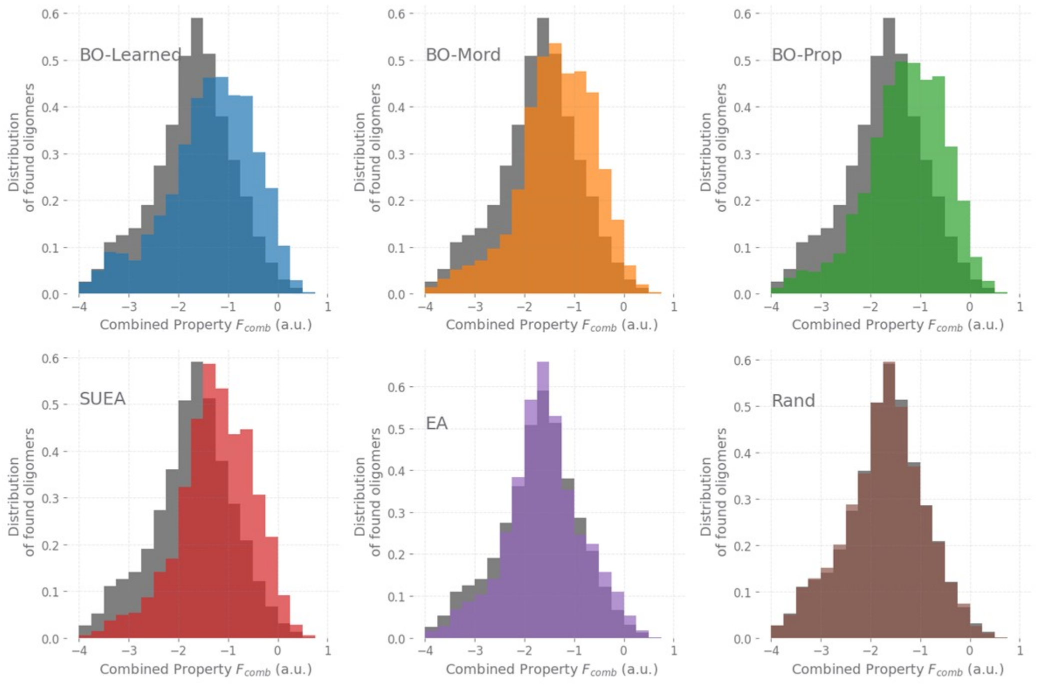

Figure S19 Distribution of the combined property of the unique oligomers suggested by the different search algorithm over 25 independent runs with different initial populations. The black shaded histogram shows the distribution of the combined property of the oligomers in the benchmark.

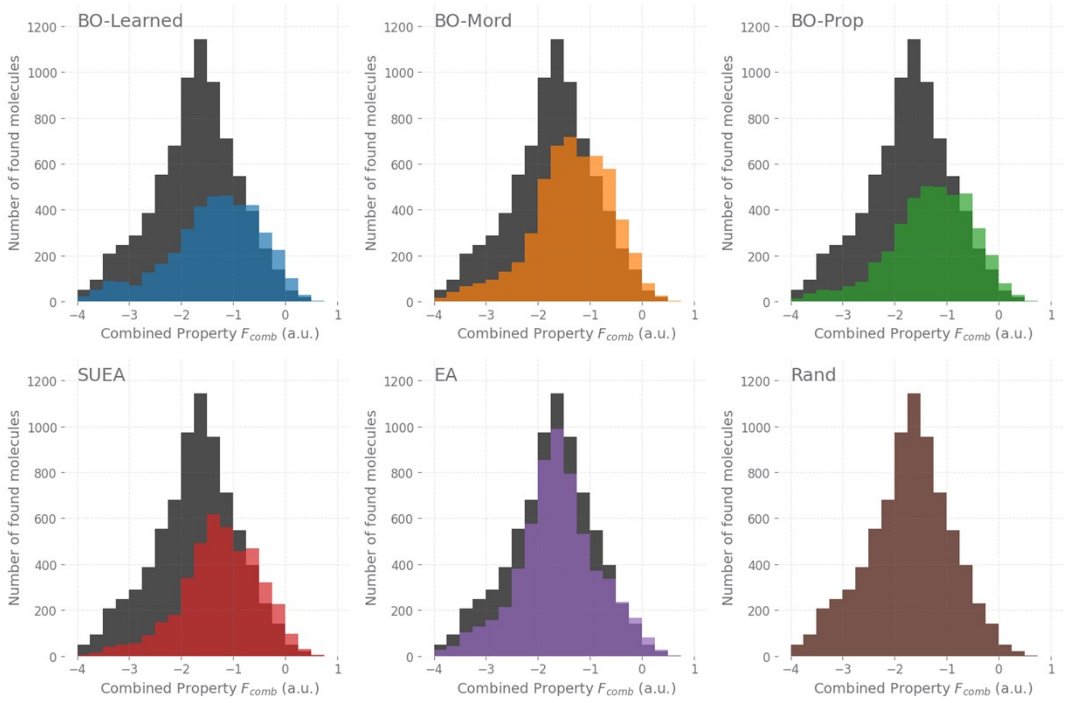

Figure S20 The number of oligomers found with target property with the different search algorithms over the benchmark search space. The panels show the distribution of the unique oligomers suggested by the search algorithms over 25 independent runs. The black shaded histogram shows the distribution of the randomly suggested elements to evaluate to compare with the different search algorithm.

b. Performance after 800 iterations

In order to investigate how the performance of the different algorithm changes when we run the search for a larger number of iterations, we have rerun the different search algorithms for 800 iterations. We limited the number to 800 for computational reasons as the BO based model require retraining a gaussian process that scales badly with the number of iterations. Figure S21 shows the performance of the search in terms of the best molecules found, the mean value of the target molecule suggested, as well as the number of top molecules found. In this case, the results are similar to those in figure 3, *BO-Learned* and *SUEA,* find the best molecules the fastest and overall suggest molecules with higher target property. We did not consider here the *BO-Mord* as the size of the representation increases the computational load considerably.

After 300 iterations, random performs better that EA in finding the molecules with the higher F_{comb}. This is related to the limited search space, where although the EA keeps predicting molecules with a higher mean F_{comb}, some of the higher performing are only accessible though mutation of very specific molecules. Hence the EA has higher chances of being stuck in an area of the chemical space, with less chances of overcoming it.

After 700 iterations, Rand is the best model at consistently finding molecules with the highest F_{comb}.Apart from the improved performance of Rand over higher number of iterations, the models perform similarly to the case in figure 3 of the paper. With increased number of iterations, the mean F_{comb} of the molecules suggested decreases for BO-learned, BO-prop and SUEA; the rate of discovery of best molecules decreases as well and gets closer to the Rand case.

We further investigate why the search algorithm perform worse in finding the top molecules after a large number of iterations as compared to a random search. In our extended

analysis of the EA and SUEA algorithms, we find that the discovery of top-performing molecules is strongly correlated with their mutational accessibility. In this context, mutational accessibility refers to how easily a target molecule can be reached from other molecules in the dataset through a series of small modifications—specifically, changes in their constituent building blocks. Within the limited chemical space of our benchmark, molecules that share at least four building blocks with a top-performing candidate are significantly more likely to guide the algorithm toward that candidate.(Figure S22 b)

For Bayesian Optimization (BO)-based models, we observe a different but related form of bias. These models are influenced by the unbalanced distribution of building blocks in the dataset. The surrogate model, trained on this skewed data, tends to favour regions of chemical space populated by frequently occurring building blocks. This bias is further reinforced by the Expected Improvement (EI) acquisition function, which prioritizes candidates that are structurally similar to many existing molecules.(figure S22 a) As a result, molecules composed of underrepresented building blocks are less likely to be selected, even if they have high potential. This is evidenced by the correlation between the likelihood of discovering top candidates and the number of molecules in the dataset that share at least four building blocks with them.

The results from running the search algorithms over 800 iterations further highlight the impact of dataset imbalance. While the benchmark dataset was generated randomly, it still exhibits structural biases common in chemical datasets. These biases increasingly affect algorithm performance as the search space coverage grows. To mitigate this issue, one could consider either (i) a balanced sampling strategy that ensures a more uniform distribution of building blocks, or (ii) a significant expansion of the benchmark dataset to dilute the effects of imbalance. However, the former approach may unfairly favour model-based algorithms over random search, while the latter would require substantially more computational resources and a redefinition of the study's scope.

Here we are focusing on the performance of the search algorithms on the limited search space of 30,000 molecules; and we allow for the exploration of more than 2% of the search space. Reaching a similar value on the full search space would mean exploring more than 10^{12} molecules, which would not be feasible. This explains the overall very low performance of Rand on the full search space (figure 4).

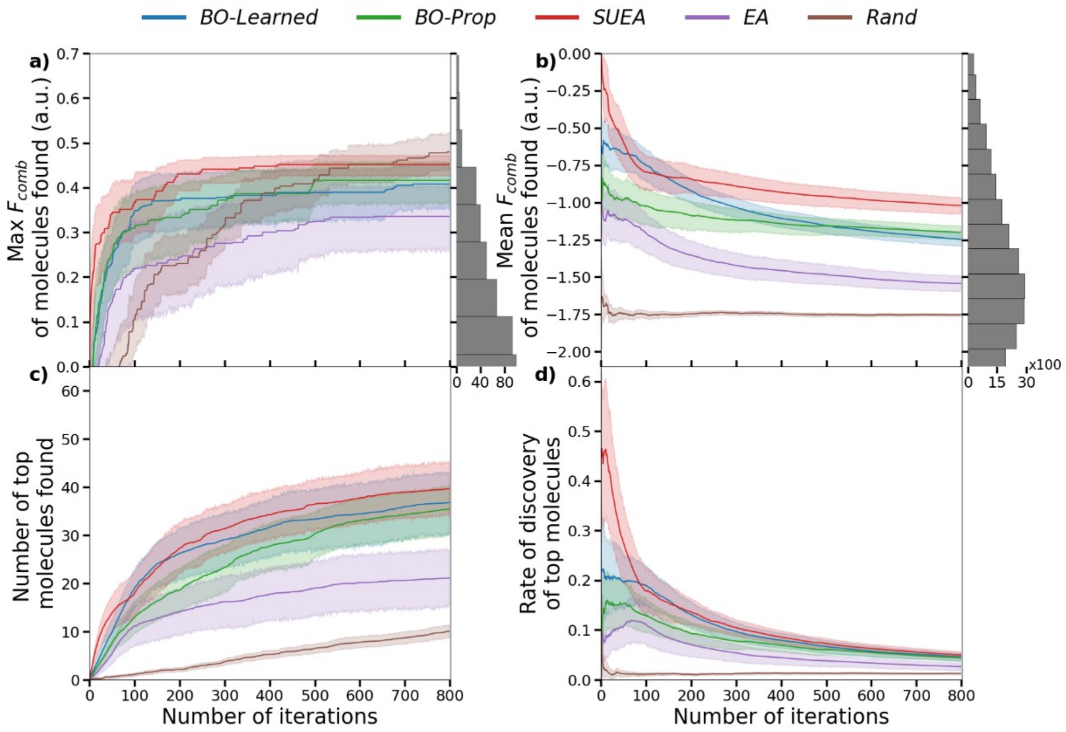

Figure S 21 Performance of the six different search approaches on the precalculated benchmark dataset of 30,000 molecules. The solid-coloured lines show the mean F_{comb} over 25 runs with different initial populations and the coloured shaded area shows the variance of the F_{comb} over those different runs; a) Maximum F_{comb} found for an oligomer up to the current iteration. The histogram on the right shows the distribution of the oligomers in the benchmark dataset; b) Mean F_{comb} of the oligomers found up to the current iteration; c) Number of oligomers in the top 1% found up to the current iteration (top 1% is 300 molecules); d) Discovery rate of the top 1% oligomers in the dataset, calculated as the (number of top molecules found)/(number of iterations).

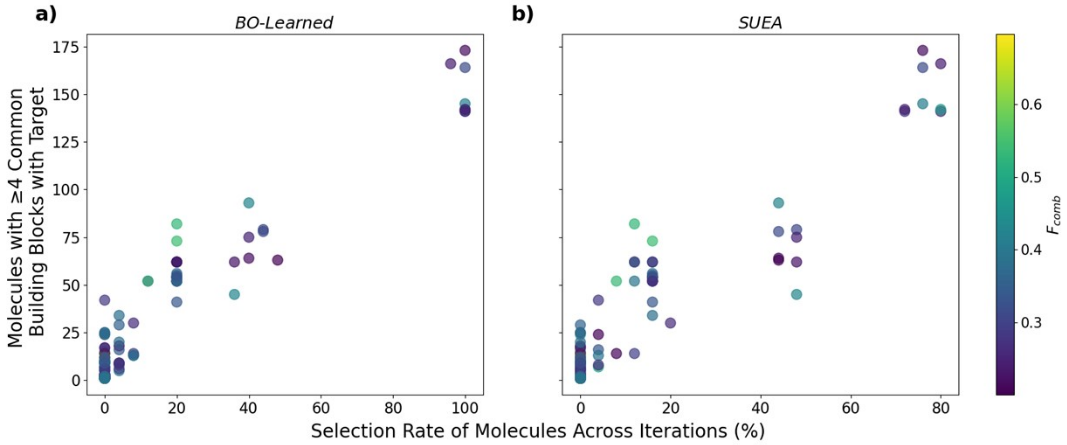

Figure S 22 Correlation between the selection rate of top-performing molecules and the number of dataset molecules sharing four building blocks with each top molecule. The selection rate is averaged over 25 independent runs after 800 iterations. Results are shown for (a) BO-Learned and (b) SUEA.

5) IMPACT OF THE TRAINING DATASET ON THE PERFORMANCE OF THE *BO_LEARNED* ALGORITHM ON THE BENCHMARK DATASET

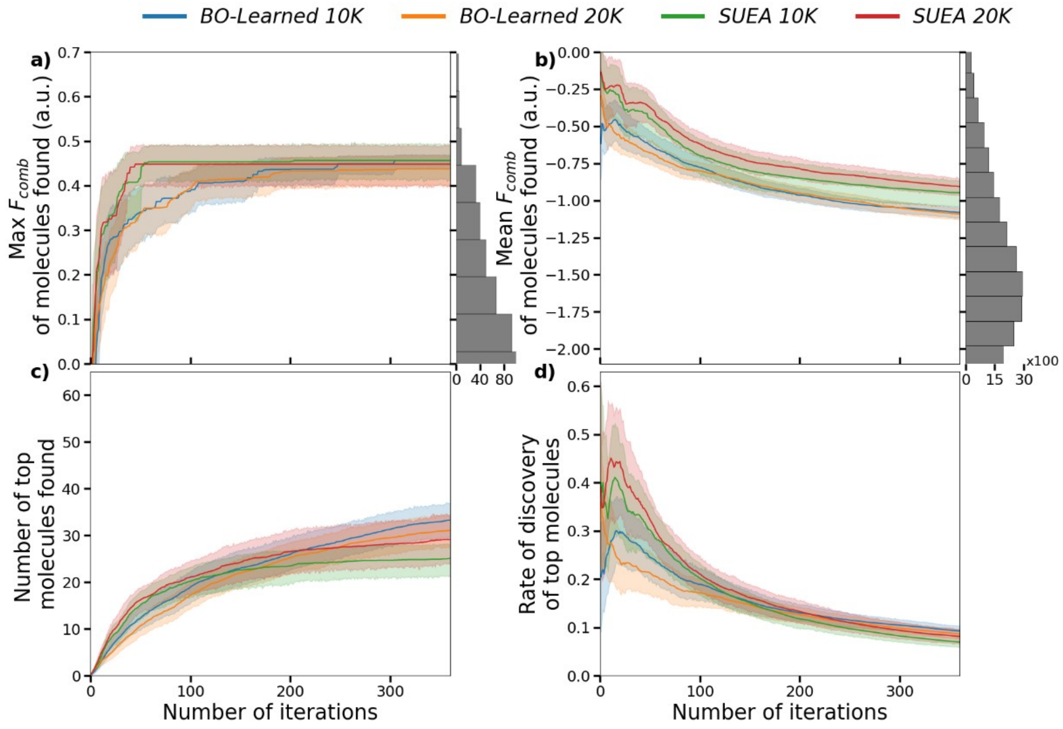

Figure S 23 Impact of the number of datapoint in the training dataset on the search algorithm performance. The performance of the search algorithms is assessed when exploring the space of precalculated oligomers (~30000 unique oligomers). The solid-coloured lines show the mean F_{comb} over 25 runs with different initial populations and the coloured shaded area shows the variance of the F_{comb} over those different runs; a) Maximum F_{comb} found in a molecule up to the current iteration. The histogram on the right shows the distribution of the molecules in the benchmark dataset; b) Mean F_{comb} of the molecules found up to the current iteration; c) Number of molecules in the top 1% found up to the current iteration (top 1% is 300 molecules); d) Discovery rate of the top 1% molecules in the dataset, calculated as the (number of top molecules found)/(number of iterations).

To assess the impact of the number of trainings datapoints, we assess the performance of the search algorithms (*SU-EA* and *BO-Learned*) with two sizes of training datasets (10k and 20k). First, we compare the performance of the models over the different dataset as shown in table S1 and Figure S24 and Figure S25. The performance of the model trained with 20,000 molecules on the test set is reduced compared to the one with 10,000. This can be related to the different training sets considered, where a different split of the dataset can result in a different performance. For the test dataset considered here, we include the top 300 molecules in the benchmark dataset, as we are interested in how the models would perform in finding the best molecules in the dataset. The split between the train and validation dataset is done randomly, with 90% of the molecules considered for the training and 10 % in the validation dataset.

The observed change in the performance of the surrogate model on the test data did not have an impact on the performance of the search algorithms (Figure S23). In this case, we find that the increase of the number of datapoints had a minor impact on the performance of the search algorithm which agrees with the minor improvement in the performance of the trained models

(table S1). Apart from the quantity of the data, it is also important to use a dataset that is diverse and representative of the search space. We have not fully explored that avenue in this work, and it would be an interesting follow up study. In the case of the *BO-Learned*, the case with 20k datapoints performed overall better than the one with 10k datapoints. For the *SU-EA*, the trend is inversed, pointing toward the limited performance of the surrogate model.

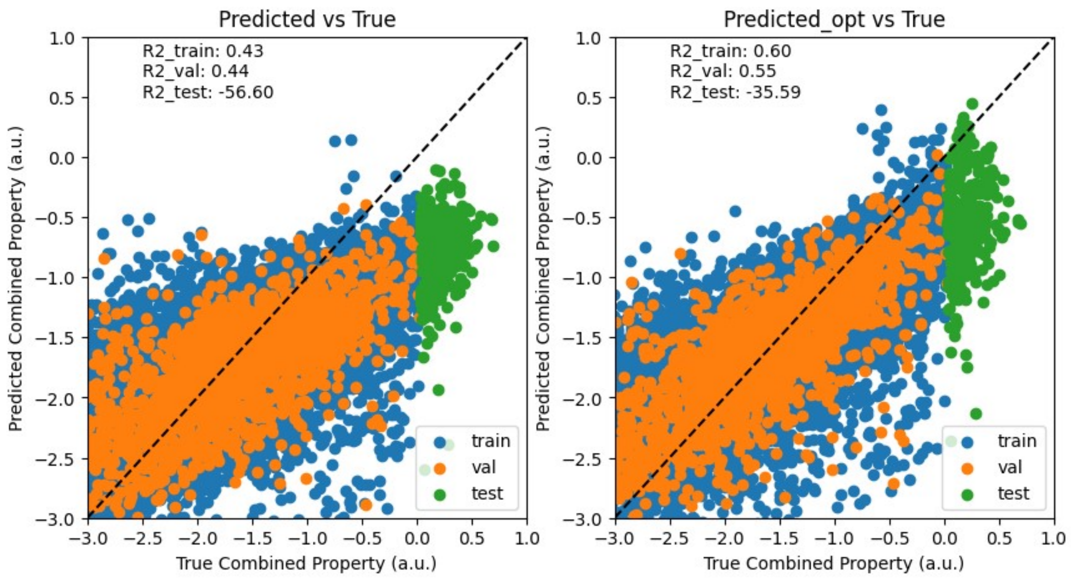

Figure S24 Performance of the pretrained model with a training dataset size of 20,000 oligomers. the right panel shows the predicted combined property using the optimised geometry of the oligomers. The left panel shows the performance of the full model considering the geometry generated using stk alone. The performance drops as we try to learn a mapping between the initial geometry from stk to the optimised one.

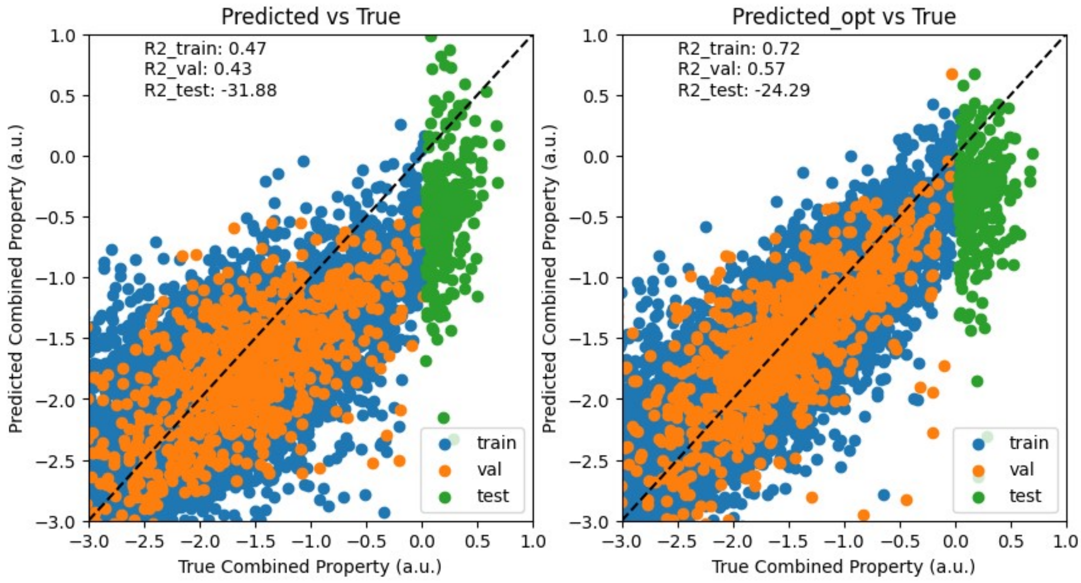

Figure S25 Performance of the pretrained model with a training dataset size of 10,000 oligomers. The right panel shows the predicted combined property using the optimised geometry of the oligomers. The left panel shows the performance of the full model considering the geometry generated using *stk* alone. The performance drops as we try to learn a mapping between the initial geometry from *stk* to the optimised one.

Table S1 performance of the pretrained model with different training dataset size. Mean average error (MAE) is the metric used in this table.

Number of oligomers in training dataset	10,000			20,000		
Metrics	MAE Train set	MAE Val set	MAE test set	MAE Train set	MAE Val set	MAE test set
Values	0.46	0.48	0.71	0.47	0.45	1.02

6) IMPACT OF CHOOSING DIFFERENT ACQUISITION FUNCTION ON THE SEARCH ALGORITHM PERFORMANCE

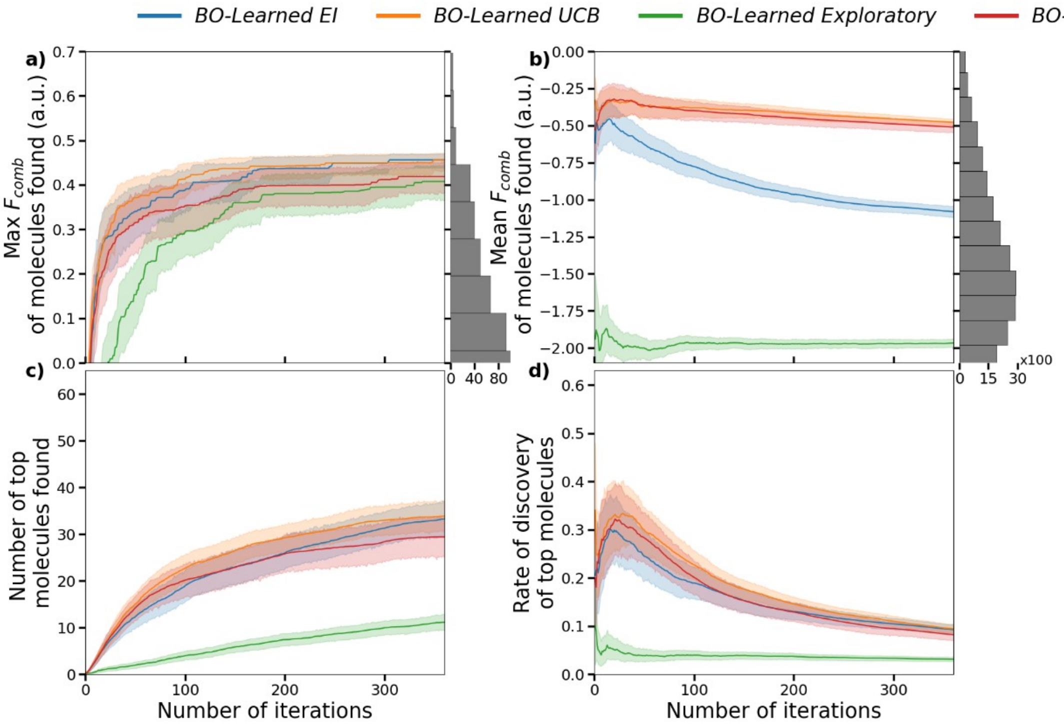

Figure S26 Impact of the acquisition function on the search algorithm performance. Here we consider four different acquisition function: Expected improvement (EI), Upper confidence bound (UCB), Exploratory (element with max uncertainty), Greedy (element with maximum of the predictive mean). Performance of the search algorithms over different metrics when exploring the space of precalculated oligomers (~30000 unique oligomers). The solid-coloured lines show the mean F_{comb} over 25 runs with different initial populations and the coloured shaded area shows the variance of the F_{comb} over those different runs; a) Maximum F_{comb} found in an oligomer up to the current iteration. The histogram on the right shows the distribution of the molecules in the benchmark dataset; b) Mean F_{comb} of the oligomer found up to the current iteration; c) Number of oligomers in the top 1% found up to the current iteration (top 1% is 300 molecules); d) Discovery rate of the top 1% oligomers in the dataset, calculated as the (number of top oligomers found)/(number of iterations).

Figure S25, shows the performance of the *BO-Learned* search algorithm over the benchmark search space with different acquisition functions. Here we consider four different acquisition function: Expected improvement (EI) which is used throughout the paper. Upper confidence bound (UCB) which is a sum of the predictive mean and the predicted uncertainty for a specific oligomer. Exploratory acquisition which only considers the predicted uncertainty and the Greedy approach which only considers the predictive mean. The Greedy and UCB acquisition perform similarly in this case, showing overall similar performance over the different metrics. The exploratory acquisition function suggests the most diverse set of oligomers but is not guided by the aim of the search to find an oligomer with high combined property. The Expected improvement in this case seems to balance exploration and exploitation in this case, which results in the highest number of top 1% oligomers found after 500 iterations.

7) RESULTS OF THE SEARCH ALGORITHM OVER THE UNRESTRICTED SPACE.

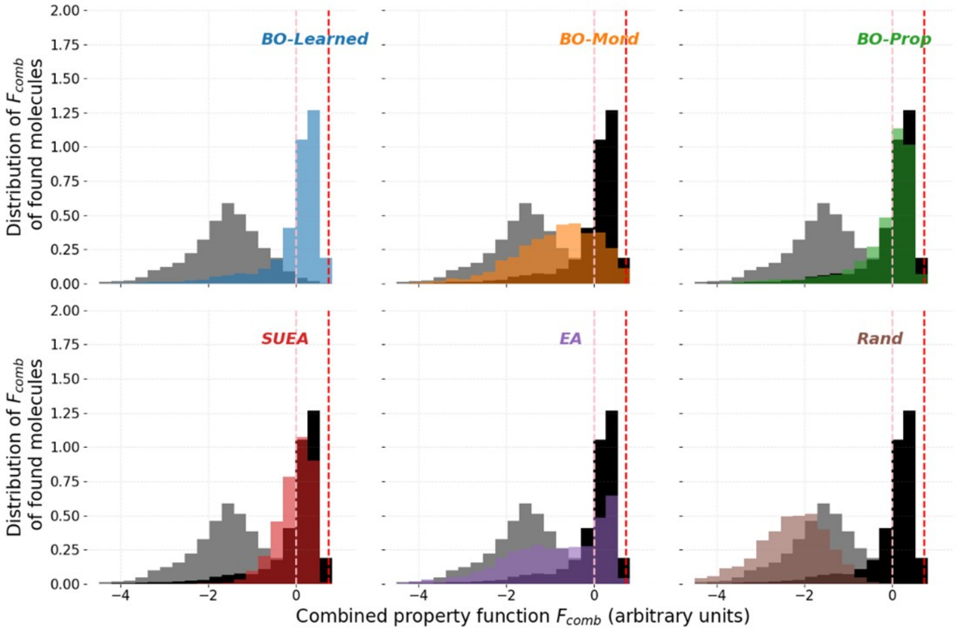

Figure S26 Distribution of the target property of the oligomers suggested by each search algorithm for the first set of runs. the first set of runs are the 50 parallel searches done after the benchmark dataset. The grey shaded area shows the distribution of the data in the benchmark dataset. The black distribution in the panels shows the distribution from the *BO-Learned* search algorithm for comparison. The pink dashed line shows the threshold to have target property above 0 and the red dashed line shows the best element in the benchmark.

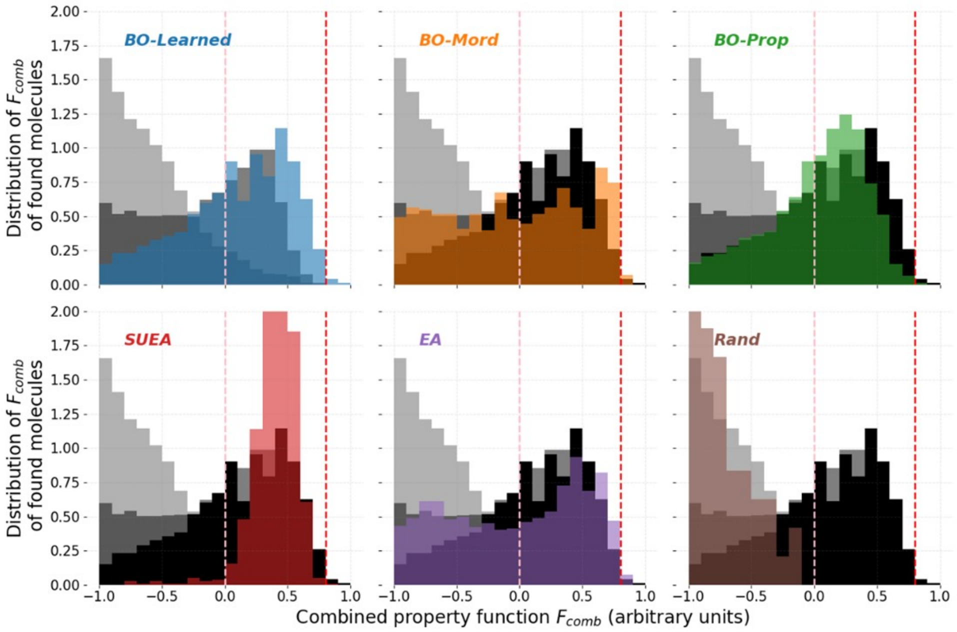

Figure S27 Distribution of the target property of the oligomers suggested by each search algorithm for the second set of runs. the second set of runs, are the 20 parallel runs of the search approach which uses the dataset generated after the first run. The light grey shaded area shows the distribution of the data in the benchmark dataset, the grey shaded area shows the distribution of the data in the dataset from the first set of runs. The black distribution in the panels shows the distribution from the *BO-Learned* search algorithm for comparison. The pink dashed line shows the threshold to be among the top 1% of the oligomers in the starting dataset and the red dashed line shows the best element in the starting dataset.

In the second set of runs with results presented in Figure S27 for the *SU-EA*, the ratio of molecules suggested with combined property increased considerably compared to the first set of runs. This can be related to the overall improved performance of the surrogate model to predict the combined property of the molecules. For the *BO-Learned*, although the surrogate model used to generate the representation performs better at predicting the combined property (as supported by the performance of the *SU-EA*), it suggested less molecules with F_{comb} above 0 as compared to its performance in the first set of runs. This could be explained by the exploratory nature of the acquisition function considered. In the context of the expected improvement acquisition function, this means that the algorithm suggests elements with higher uncertainty rather than overall higher predicted combined property value. In terms of finding molecules that are either closer to the best of the current dataset or even better, the *BO-Mord* and the simple *EA*, show the best performance overall. In this case, the *EA* finds 7 molecules with higher combined property than the ones in the dataset, whereas the BO-*Learned* only finds 4 new molecules. These results confirm the initial observation, that a model that has more information about the space, fails to find elements better than the dataset it has been trained on.

8) COMPUTATION TIME OF SEARCHING OVER UNRESTRICTED SPACE.

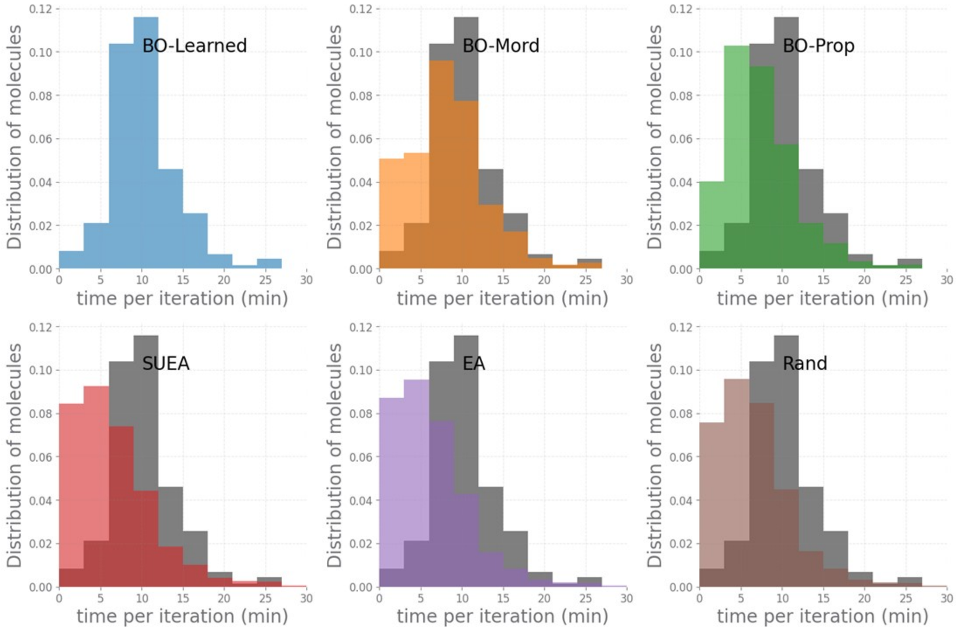

Figure S29 Distribution of time per iteration for the different search algorithms. The distribution here is over the oligomers found through the second set of runs over the unrestricted space.

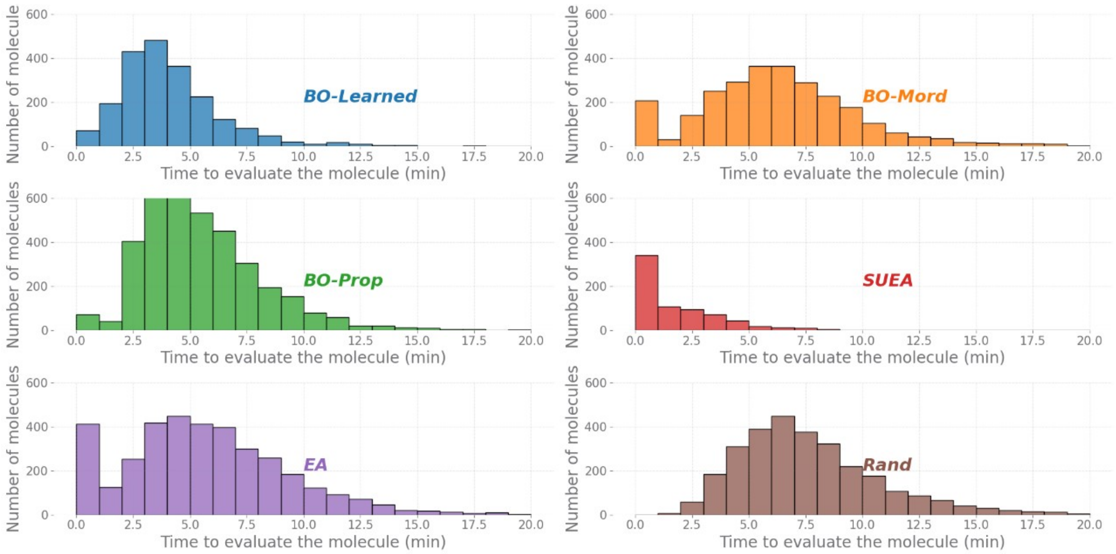

Figure S30 distribution of the time needed to evaluate an oligomer using the evaluation function.

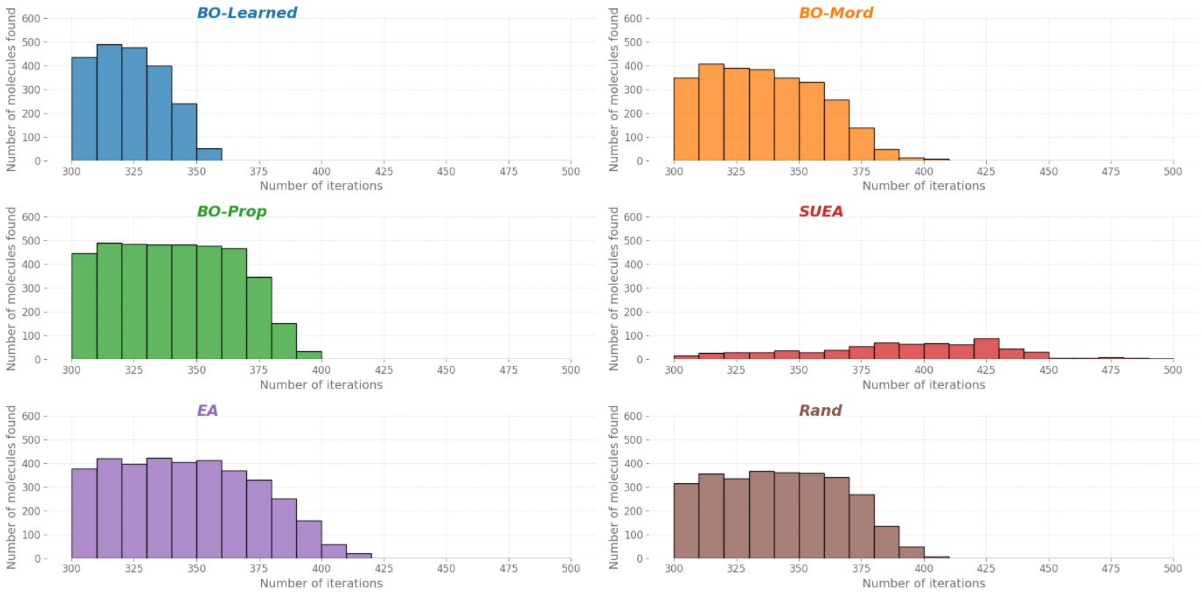

Figure S31 New oligomers found over the set of runs across different number of iterations. Here we show the sum of the oligomers suggested by the parallel runs at a specific iteration. The change in the number of oligomers found with iteration is due to both a change in the number of runs that reached a certain number of iteration as well as the possibility of the parallel runs to suggest the same molecules.

9) DFT/TDDFT RESULTS

In this section, we show the results of calculating the properties of interest, namely the first excited state energy and oscillator strength as well the ionisation energy of the most promising molecules suggested by the different algorithms using a higher level of theory than XTB/sTDA. We consider the top 5 molecules suggested by the first run and the top 5 molecules suggested by the second run on the full search space. The structures of the molecules considered are shown in figure S32.

To calculate the properties of the molecules, we start with the geometries generated using the Supramolecular Toolkit (STK). These geometries are then optimized using the GFN2-xTB method, which is suitable for rapid and accurate electronic structure calculations of large molecular systems. Next, we use Gaussian to optimize the molecules in vacuum employing the ωB97XD functional with the 6-31G* basis set. The ωB97XD functional is chosen for its ability to accurately describe weak interactions and its proven correlation with experimental data, while the 6-31G* basis set provides a good balance between computational cost and accuracy.[17]

For ionization potential (IP) calculations, we optimize the geometry of the molecules in their cationic state. The IP is estimated by calculating the difference in total energy between the ground state geometry and the optimized cation geometry. To determine the excited state energy and oscillator strength, we perform time-dependent density functional theory (TD-DFT) calculations on the molecule in its ground state optimized geometry.

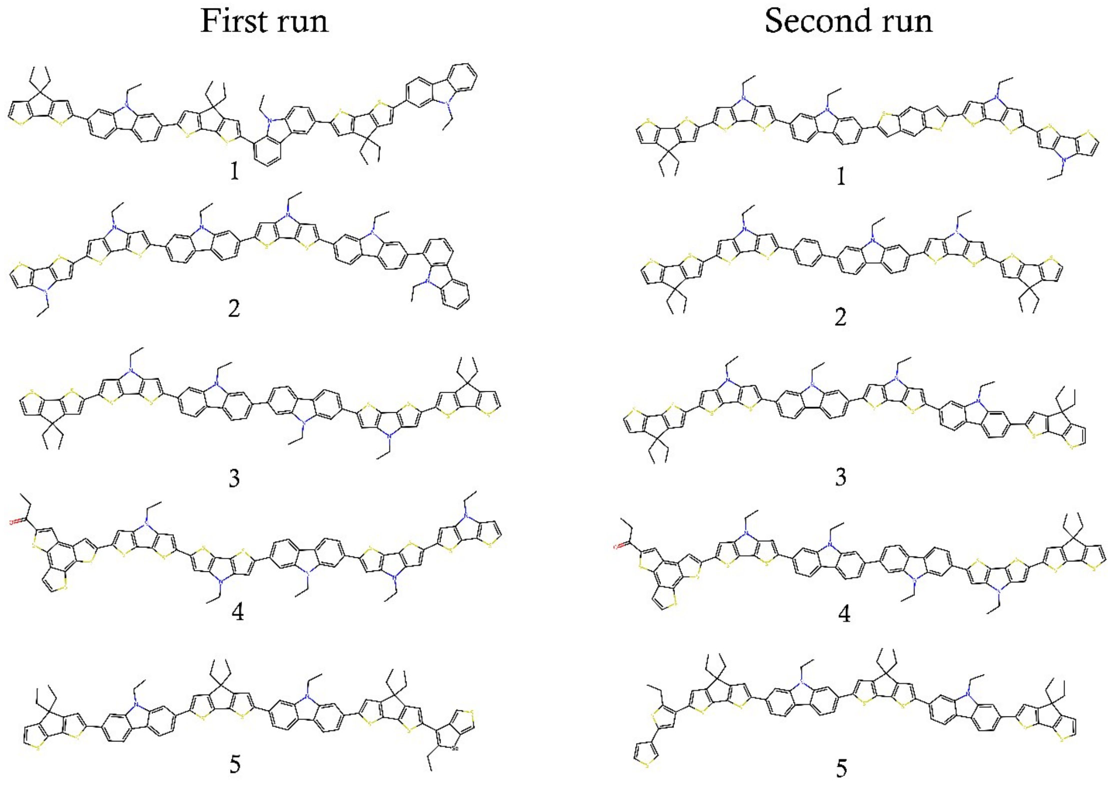

Figure S 32 Best 5 molecules after each run considered for the DFT/TDDFT calculation. The labels shown here are the ones used in table S2.

The comparison of the properties of interest for the ten selected molecules calculated at the XTB/sTDA level and the DFT/TDDFT level is shown in table S2. First most of the identified molecules using XTB/sTDA, are promising candidates for the application considered.

Meaning the computed properties using DFT/TDDFT suggest that they would be good candidates as donor molecules with Y6. Specifically, they all show a considerably higher oscillator strength than other molecules considered for optoelectronic applications.[18] The differences observed between the calculated properties using the two methods is expected, however we observe that both E_{s1} and IP are within 0.3-0.5 eV of each other, confirming that XTB/sTDA is indeed a good proxy to compute the properties considered. However, a more thorough analysis of the correlation between the two levels of theory, along with experimental validation, is necessary to further confirm the potential of the proposed method for discovering new molecules suitable for real-world applications.

Table S 2 Comparison of the XTB/sTDA and the DFT/TDDFT calculated properties of the 5 top performing molecules in each run.

	XTB/sTDA			DFT/TDDFT		
Id	E_{S1} (eV)	IP (eV)	$f_{osc,1}$	E_{S1} (eV)	IP (eV)	$f_{osc,1}$
Second Run						
1	3.02	5.48	8.82	2.81	5.53	3.19
2	2.99	5.50	7.91	2.87	5.82	3.43
3	3.00	5.46	7.77	2.94	5.86	4.17
4	2.97	5.51	7.61	2.79	5.49	3.59
5	2.99	5.49	7.09	3.39	6.44	5.41
First Run						
1	3.02	5.51	6.87	3.37	6.12	4.69
2	3.06	5.50	7.32	3.39	5.97	4.79
3	3.02	5.45	7.35	2.82	5.78	4.48
4	2.96	5.44	7.53	3.30	5.80	5.51
5	2.98	5.52	6.55	3.53	5.89	4.96

10) REFERENCES

1. B. L. Greenstein and G. R. Hutchison, *Journal of Physical Chemistry C*, 2023, **127**, 6179-6191, DOI: 10.1021/acs.jpcc.3c00267.
2. L. Turcani, A. Tarzia, F. T. Szczypinski and K. E. Jelfs, *J Chem Phys*, 2021, **154**, 214102, DOI: 10.1063/5.0049708.
3. C. Herrera-Acevedo, C. Perdomo-Madrigal, J. A. de Sousa Luis, L. Scotti and M. T. Scotti, in *Drug Target Selection and Validation*, eds. M. T. Scotti and C. L. Bellera, Springer International Publishing, Cham, 2022, pp. 1-24 DOI: 10.1007/978-3-030-95895-4_1.
4. S. R. Krishnan, N. Bung, R. Srinivasan and A. Roy, *Journal of Molecular Graphics and Modelling*, 2024/06/01, **129**, DOI: 10.1016/j.jmgm.2024.108734.
5. R. Guo, *Communications of the Acm*, 2017, **60**, 43-47, DOI: 10.1145/3052937.
6. C. Bannwarth, S. Ehlert and S. Grimme, *J Chem Theory Comput*, 2019, **15**, 1652-1671, DOI: 10.1021/acs.jctc.8b01176.
7. S. Grimme and C. Bannwarth, *J Chem Phys*, 2016, **145**, 054103, DOI: 10.1063/1.4959605.
8. J. R. Gardner, G. Pleiss, D. Bindel, K. Q. Weinberger and A. G. Wilson, *Advances in Neural Information Processing Systems 31 (Nips 2018)*, 2018, **31**, DOI: 10.48550/arXiv.1809.11165.
9. M. Balandat, B. Karrer, D. R. Jiang, S. Daulton, B. Letham, A. G. Wilson and E. Bakshy, *arXiv*, 10/2019, DOI: 10.48550/arXiv.1910.06403.
10. H. Moriwaki, Y. S. Tian, N. Kawashita and T. Takagi, *J Cheminform*, 2018, **10**, 4, DOI: 10.1186/s13321-018-0258-y.
11. S. C. Liu, W. T. Du, Y. J. Li, Z. X. R. Li, Z. L. Zheng, C. R. Duan, Z. M. Ma, O. Yaghi, A. Anandkumar, C. Borgs, J. Chayes, H. Y. Guo and J. Tang, *Adv Neur In*, 2023, DOI: 10.48550/arXiv.2306.09375.
12. K. T. Schutt, H. E. Sauceda, P. J. Kindermans, A. Tkatchenko and K. R. Muller, *J Chem Phys*, 2018, **148**, 241722, DOI: 10.1063/1.5019779.
13. J. Terven, D. M. Cordova-Esparza, A. Ramirez-Pedraza, E. A. Chavez-Urbiola and J. A. Romero-Gonzalez, *arXiv*, 2023, 2307.02694, DOI: 10.48550/arXiv.2307.02694.
14. A. P. Bento, A. Hersey, E. Félix, G. Landrum, A. Gaulton, F. Atkinson, L. J. Bellis, M. De Veij and A. R. Leach, *J Cheminformatics*, 2020, **12**, DOI: 10.1186/s13321-020-00456-1.
15. Greg Landrum, Paolo Tosco, Brian Kelley, g. sriniker, Nadine Schneider and R. Riccardo Vianello, Andrew Dalke, Brian Cole, AlexanderSavelyev, Matt Swain, Samo Turk, Dan N, Alain Vaucher, Eisuke Kawashima, Maciej Wójcikowski, Daniel Probst, guillaume godin, , *Computer Program*, 2023, DOI: 10.5281/zenodo.11102446.
16. D. Bajusz, A. Racz and K. Heberger, *J Cheminform*, 2015, **7**, 20, DOI: 10.1186/s13321-015-0069-3.
17. X. Wu, X. Xie and A. Troisi, *Journal of Materials Chemistry C*, 2024, **12**, 18886-18892, DOI: 10.1039/d4tc03511a.
18. J. Yan, X. Rodriguez-Martinez, D. Pearce, H. Douglas, D. Bili, M. Azzouzi, F. Eisner, A. Virbule, E. Rezasoltani, V. Belova, B. Dorling, S. Few, A. A. Szumska, X. Hou, G. Zhang, H. L. Yip, M. Campoy-Quiles and J. Nelson, *Energy Environ Sci*, 2022, **15**, 2958-2973, DOI: 10.1039/d2ee00887d.
